# Structural and Thermal Evolution of an Infant Subduction Shear Zone: Insights From Sub‐Ophiolite Metamorphic Rocks Recovered From Oman Drilling Project Site BT‐1B

**DOI:** 10.1029/2021JB021702

**Published:** 2021-12-04

**Authors:** Alissa J. Kotowski, Mark Cloos, Daniel F. Stockli, Eytan Bos Orent

**Affiliations:** ^1^ Department of Geological Sciences Jackson School of Geosciences University of Texas at Austin Austin TX USA; ^2^ Department of Earth and Planetary Sciences McGill University Montréal QC Canada; ^3^ Now at Department of Earth Sciences Utrecht University Utrecht The Netherlands; ^4^ Now at Department of Geosciences University of Arizona Tucson AZ USA

**Keywords:** subduction, exhumation, ophiolite, metamorphic sole, amphibolite, Oman

## Abstract

Subduction interface thermal structure changes drastically within the first few million years of underthrusting (i.e., *subduction infancy*). Metamorphic soles beneath ophiolites record snapshots of dynamic conditions and mechanical coupling during subduction infancy. Beneath the Samail Ophiolite (Oman), the sole comprises structurally higher high‐temperature (HT) and lower low‐temperature (LT) units. This inverted metamorphic gradient has been attributed to evolving metamorphic Pressure‐Temperature (P‐T) conditions during infancy; however, peak P‐T and timing of LT sole subduction are poorly constrained. Oman Drilling Project core BT‐1B sampled the base of the ophiolite in a location lacking the HT sole. Metasedimentary and meta‐mafic samples collected from 104 m of core reveal that the LT sole subducted to similar peak P as HT rocks preserved elsewhere in Oman, but experienced ∼300°C lower peak T. Prograde fabrics record Si‐in‐phengite and amphibole chemistries consistent with peak P‐T of ∼7–10 kbar and ∼450–550°C in the epidote‐amphibolite facies. Retrograde fabrics record a transition from near‐pervasive ductile to localized brittle strain under greenschist facies conditions. Titanite U‐Pb ages (*n* = 2) constrain timing of peak LT sole subduction to ∼91 Ma (post‐dating initial HT sole subduction by ∼12–13 Myr) and dynamic retrogression through ∼90 Ma. Combined with existing geo/thermo‐chronology, our results support a model of protracted subduction and accretion while the infant subduction zone experienced multi‐phase, slow‐fast‐slow cooling. Temporal overlap of HT sole cooling (rehydration?) and ophiolite formation suggests that cooling may lead to interface weakening, facilitating upper‐plate extension and spreading. The LT sole formed in a rapidly‐refrigerating forearc after ophiolite formation and may reflect the transition to self‐sustaining subduction.

## Key Points

- The low‐temperature metamorphic sole beneath the Samail Ophiolite records subduction and return flow before juxtaposition with the ophiolite
- High sampling density of the 104 m‐thick core reveals it comprises three subunits that record continuous and stepwise changes in peak P‐T
- Low‐T sole rocks reached similar peak P as the high‐T sole but colder peak T (7–10 kbar, 500°C) and witnessed rapid interface refrigeration

## 1 Introduction

During *subduction infancy,* or the first few million years following subduction initiation (Stern & Bloomer, 1992), stable metamorphic rock types and depths of interplate coupling evolve in response to continuous changes in thermal structure (e.g., Agard et al., 2016; Hacker et al., 2003; Soret et al., 2017). Mechanical weakening and progressive strain localization must occur along the interface in order to facilitate slab‐mantle coupling and large‐scale convection (e.g., Coltice et al., 2017; Gurnis et al., 2004; Wada et al., 2008; Wada & Wang, 2009). However, even the most rigorous dynamically‐consistent models of subduction initiation have yet to reproduce observed thermal characteristics during the earliest stages (e.g., Holt & Condit, 2021), which points to a disconnect in our understanding of how subduction zone strength and temperature coevolve during subduction infancy. Furthermore, rates of interface cooling and consequences of cooling for metamorphic mineral stability and rock rheology are poorly constrained. Metamorphic rocks that formed during subduction infancy record Pressure‐Temperature‐Deformation‐time (P‐T‐D‐t) trajectories that can provide direct constraints on how a subduction zone evolves toward, and eventually achieves, a self‐sustaining state.

**Figure 1:**
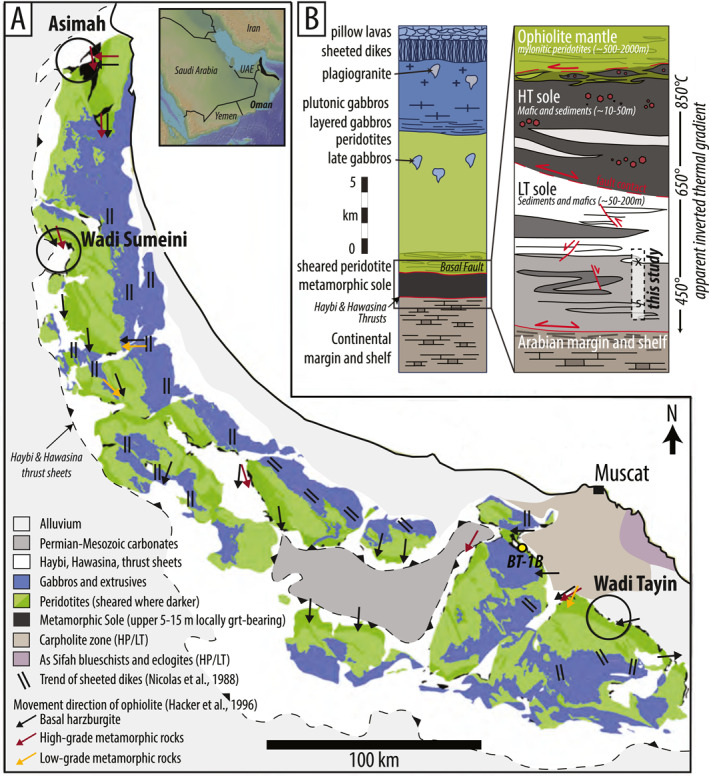
(a) Geologic map of the Samail ophiolite, modified from Agard et al. (2016). OmanDP Site BT‐1B is shown in the yellow circle. Large black circles correspond to the three best‐studied areas where metamorphic sole rocks are exposed. Orientation of sheeted dikes and movement directions (mineral and stretching lineations) compiled by Hacker et al. (1996) after Nicolas et al. (1988) (b) Schematic ophiolite sequence, modified from Hacker et al. (1996). Inset shows the metamorphic sole. In this idealized sketch, the 100 m of metamorphic rocks in core BT‐1B are marked by the dashed gray box labeled “this study”. The core records a lithologic change (marked by the “x”) and a possible structural discontinuity (marked by “s”). Red double‐headed arrows indicate likely reactivation of thrusts as normal faults.

Tectonometamorphic evidence for interface behavior during subduction infancy is preserved in the geologic record as thin (∼10–500 m) zones of metamorphosed oceanic crust and sedimentary rocks beneath ophiolites, called *metamorphic soles* (Dewey, 1976; Wakabayashi & Dilek, 2000; Williams & Smyth, 1973). For example, in Newfoundland (Dewey & Casey, 2013; Williams & Smyth, 1973), Turkey (Çelik et al., 2011; Wakabayashi & Dilek, 2000), the Philippines (Encarnación et al., 1995), New Caledonia (Cluzel et al., 2001; Cluzel et al., 2012), and Oman (Ghent & Stout, 1981; Hacker & Gnos, 1997) (Figure 1), large‐slab ophiolites have extensive exposures of metamorphic soles that record hot temperature‐depth trajectories for ∼5–15 Myr after subduction initiation. These soles consist of a thin (∼m to 10's of m) structurally higher, garnet‐amphibolite to granulite facies high‐temperature section (“HT sole”) and a thicker (∼10's to several 100's m) structurally lower, upper‐greenschist to lower‐amphibolite facies low‐temperature section (“LT sole”) (Figure 1b). The apparent inverted metamorphic gradient was originally attributed to downward conductive heating from a hot mantle wedge, sometimes described as the “ironing effect” (e.g., Gnos & Peters, 1992; Hacker & Mosenfelder, 1996; Jamieson, 1981). Several researchers have argued that HT and LT sections are distinct tectonic units that metamorphosed under discretely different P‐T conditions (most importantly, different depths) and were progressively accreted before ophiolite emplacement (Agard et al., 2016; Agard et al., 2020; Cowan et al., 2014; Searle & Cox, 1999; Soret et al., 2017). However, other work points to semi‐continuous changes in peak P‐T along a smooth geotherm throughout the structural section (Garber et al., 2020; Gnos, 1998; Hacker & Mosenfelder, 1996). Further structural, petrologic, and geochronologic data are needed to distinguish between these models (or lend support to both) and better quantify rates and conditions of interface cooling as infant subduction zones thermally “mature”.

Beneath the Samail Ophiolite in Oman, metamorphic sole rocks crop out as discontinuous lenses along its entire ∼550 km length (Figure 1). In this study, we present new structural, petrologic, and geochronologic data that refine the deformation and metamorphism history of LT sole rocks that were sampled at Oman Drilling Project site BT‐1B (Figure 1). In this core, a 40 cm‐thick cataclastic fault zone separates 195 m of hydrated (serpentinite) and carbonated (listvenite) ultramafic rocks from a 104‐m thick section of LT sole. We present data from a densely sampled suite of rocks that span the entire 104 m of core with primary goals to distinguish prograde from retrograde fabrics, constrain corresponding P‐T conditions, and U‐Pb date syn‐kinematic, prograde and retrograde titanite crystallization. This work provides new insight into the tectonic relationship between the HT and LT sole slivers, and has implications for rates of refrigeration and evolving interface dynamics during subduction infancy.

## 2 Geologic Context

The Samail Ophiolite, with an outcropping area of ∼17,000 km^2^, is the most complete and best‐exposed subaerial complex of oceanic crust and upper mantle in the world. Spanning ∼550 km N–S and ∼100–150 km E–W along the coastline of Oman and the United Arab Emirates, the ophiolite comprises ∼4–7 km of oceanic crustal rocks atop ∼8–12 km of mantle peridotite (Allemann, 1972; Lippard et al., 1986; Nicolas et al., 1988) (Figure 1). Zircon U‐Pb ages from plagiogranites in upper‐level gabbros constrain ophiolite crystallization between ∼96.4–95.5 Ma along its entire length (Rioux et al., 2012; Rioux et al., 2021, 2013; Tilton et al., 1981). More specifically, Rioux et al. (2021) coupled U‐Pb zircon dates with Sm‐Nd isotopes to distinguish two main phases of ophiolite formation: primarily decompression‐related V1 moist‐MORB magmatism between 96.1–95.6 Ma, followed by V2 water‐fluxed mantle melting from 95.6–95.2 Ma.

At the base of the ultramafic portion of the ophiolite, a ductile shear zone and/or fault contact juxtaposes the lithospheric mantle with discontinuous km‐scale lenses of metamorphic sole rocks up to ∼200 m thick (Hacker and Mosenfelder, 1996, black lenses in Figure 1a). The ophiolite‐sole package rests structurally atop the Haybi and Hawasina formations, which comprise a weakly metamorphosed and variably imbricated section of Arabian continental proximal and distal margin (Searle & Malpas, 1980). The western leading edge of the ophiolite appears to have been transported as much as 250 km during underthrusting of the Arabian continental margin in the Upper Cretaceous (Glennie, 1974). Offset along individual post‐metamorphic out‐of‐sequence thrusts and normal faults are up to several kilometers, but large portions of the ophiolite have remained remarkably intact (Nicolas et al., 1988).

While ophiolites are recognized as slabs of oceanic crust and upper mantle, the geodynamic setting for their formation is debated. Many workers have long posited that the Samail Ophiolite formed at a mid‐ocean spreading ridge (Boudier et al., 1985; Hopson et al., 1981), on the basis of immobile trace element compositional data from older basalts and gabbros (Godard et al., 2006). In this scenario, emplacement‐related thrusting is initiated along the ridge axis (Agard et al., 2007; Agard et al., 2014; Duretz et al., 2016; Hacker, 1991). In contrast, a competing model argues that the Samail Ophiolite formed above a subducting plate, in a supra‐subduction zone setting (Alabaster et al., 1982; MacLeod et al., 2013; Searle & Cox, 2002; Searle & Malpas, 1980, 1982). This model is supported by younger ophiolitic lavas characterized by up‐section decreasing abundances of incompatible elements and increasing LILEs relative to N‐MORB (Alabaster et al., 1982; Belgrano & Diamond, 2019; Ishikawa et al., 2002; Pearce et al., 1981). MacLeod et al. (2013) went further using petrologic models of immobile major elements and argued that even the relatively early axial volcanics and dike sequences exhibit fractionation trends that require water concentrations significantly higher than any N‐MORB (see also Belgrano and Diamond, 2019). They concluded that the magmatism was comparable to modern intra‐oceanic forearc/proto‐arc spreading systems. Furthermore, zircon U‐Pb geochronology and Sm‐Nd isotopes presented by Rioux et al. (2021) suggest a similar temporal and geochemical magmatic evolution to the Marianas, further strengthening the supra‐subduction zone interpretation. In the supra‐subduction model, the ophiolite formed and then was emplaced atop the Arabian continental margin as the NE‐dipping subduction zone was jammed during continental subduction (Lippard et al., 1986; Searle & Cox, 1999; Searle & Malpas, 1980, 1982).

### 2.1 Origin and Evolution of the Metamorphic Sole

Structural and geochemical constraints from sub‐ophiolite metamorphic rocks provide further insight into the geodynamic processes that led to formation of the Samail Ophiolite. In Oman and the UAE, similar to other global ophiolite exposures, the metamorphic sole is in tectonic contact against variably sheared and partially serpentinized basal mantle peridotites. The contact is mostly exposed as a brittle fault zone, although locally the HT sole‐banded peridotite (mantle) contact is a ductile shear zone (e.g., Sumeini and Asimah; Searle and Malpas, 1982; Soret et al., 2017). The HT unit comprises meters‐to‐tens of meters‐thick garnet‐clinopyroxene amphibolites (i.e., retrogressed granulites; Ambrose et al., 2021), grt‐ or cpx‐only amphibolites, and grt‐cpx‐free amphibolites, locally containing lenses of crystallized partial melt (Hacker & Mosenfelder, 1996; Soret et al., 2017). The HT unit is structurally above a LT unit of tens‐to‐several hundreds of meters‐thick greenschist to lower‐amphibolite facies metamorphosed sedimentary rocks and basalts (Bucher, 1991; Hacker & Mosenfelder, 1996; Searle & Malpas, 1980, 1982; Searle & Cox, 2002) (Figure 1b). Trace element geochemistry of the HT sole suggests the protoliths were both E‐MORB and N‐MORB that were not genetically related to lavas in the ophiolite (Ishikawa et al., 2002; Searle & Cox, 2002). This seemingly rules out the simple model of subduction initiation at a mid‐oceanic spreading ridge.

Estimates of metamorphic conditions from garnet‐pyroxene thermometry, petrologic phase equilibria, and phase equilibria modeling indicate the HT sole reached ∼750–850°C and 10–14 kbar (Cowan et al., 2014; Ghent & Stout, 1981; Gnos, 1998; Hacker & Gnos, 1997; Hacker & Mosenfelder, 1996; Searle & Cox, 2002; Searle & Malpas, 1982; Soret et al., 2017). Pressure estimates of 10–14 kbar are equivalent to ∼35–50 km depth and are ∼2–3× times greater than the ∼12–20 km exposed thickness of the ophiolite section in Oman. This is considered strong evidence that the HT sole rocks were subducted and partially exhumed prior to arriving at their present structural location against the ophiolite (e.g., Cowan et al., 2014; Hacker & Gnos, 1997; Searle & Cox, 2002; Soret et al., 2017).

High‐precision U‐Pb zircon crystallization ages from ophiolite plagiogranites and leucocratic lenses in HT sole garnet amphibolites are nearly contemporaneous, and mostly support the interpretation that sole metamorphism was synchronous or slightly post‐dated (but in some cases slightly pre‐dated by a few 100 kyr) ophiolite formation (e.g., 95.3 ± 0.2 Ma (ophiolite) vs. 94.48 ± 0.23 Ma (sole) from Warren et al., 2005; 96.12–95.5 Ma (ophiolite) vs. 96.16 ± 0.022 and 94.82 ± 0.035 Ma (sole) from Rioux et al., 2016). Recently, Garber et al. (2020) presented Lu‐Hf garnet crystallization ages, along with zircon (TIMS and LA‐ICP‐MS), monazite (LA‐ICP‐MS), and titanite (LA‐ICP‐MS) U‐Pb ages, from a garnet‐ and mica‐rich metasedimentary rock collected ∼12 m beneath the Basal Thrust at Wadi Tayin (Figure 1). These authors concluded that this sample underwent prograde heating and metamorphism as early as 98.7–94.1 Ma (U‐Pb zircon and monazite) and reached peak conditions (∼7.5 ± 1.2 kbar and 665 ± 32°C) at ∼93 Ma (Lu‐Hf garnet). The interpretation that Lu‐Hf ages capture the timing of peak metamorphism is justified because the majority of the Lu is concentrated in garnet rims, and according to their thermobarometric modeling, the garnet rims grew at peak P‐T conditions. However, Guilmette et al. (2018) presented garnet Lu‐Hf ages for three meta‐mafic samples (note the different bulk composition) from the uppermost HT sole at Wadi Tayin and Wadi Sumeini localities that yielded significantly older ages of ∼103–104 Ma. These data suggest that HT sole metamorphism pre‐dated igneous crystallization of the ophiolitic crust by ∼8 Myr. Their three samples yielded identical ages (within error), despite having very different Lu zoning profiles. Furthermore, trace element zoning patterns and isochrons with low MSWDs indicate low geologic scatter and rapid crystal growth. Therefore, these Lu‐Hf ages are likely volumetrically averaged garnet growth ages from subsolidus nucleation to peak metamorphism and partial melting. In light of the older garnet ages, Garber et al. (2020) concluded that their metasedimentary sample reached peak conditions either 2 or 11 Myr (whether the uppermost meta‐mafic HT sole is roughly the same age as or older than the ophiolite, respectively) after the structurally higher meta‐mafic rocks, indicative of: (a) younging peak metamorphism away from the thrust and (b) a dominant phase of metamorphism post‐dating ophiolite formation. If the ∼104 Ma garnet ages are accurate, then extreme thermal gradients were maintained along the nascent subduction zone between ∼104–93 Ma (Garber et al., 2020) and the U‐Pb zircon ages from the HT sole could record the timing of partial melt crystallization rather than peak HT sole metamorphism (Rioux et al., 2016).

While the garnet‐bearing HT sole has received lots of attention, the metamorphic and deformational history of the more voluminous LT sole has been largely neglected. In Oman, early studies suggested that the LT sole was a “monometamorphic” unit, freezing in a single greenschist facies event during emplacement beneath the HT unit (Allemann, 1972; Ghent & Stout, 1981; Glennie, 1974). However, observations from Asimah (Bucher, 1991) and from Wadi Sumeini and Wadi Tayin (Hacker & Mosenfelder, 1996), suggested that the LT sole reached epidote‐amphibolite facies prior to a near‐pervasive greenschist facies overprint. Amphibole‐plagioclase thermobarometry and Raman spectroscopy on carbonaceous material suggested peak P–T of ∼4.5–5.5 kbar and ∼450–500°C and greenschist facies retrogression at ∼340–380°C (Bucher, 1991; Gnos, 1998; Hacker & Mosenfelder, 1996; Soret et al., 2017). The timing of metamorphism in the LT sole is poorly constrained, but white mica Ar/Ar and biotite K/Ar ages bracket cooling through ∼450–350°C (or (re‐)crystallization) between ∼95–90 Ma (Gnos & Peters, 1992; Guilmette et al., 2018; Hacker, 1994; Hacker et al., 1996). Other than the garnet Lu‐Hf ages, these Ar ages are only 3–5 million years younger than ages reported for the HT sole.

### 2.2 Recovery of LT Sole Rocks During the OmanDP and Key Objectives at Site BT‐1B

**Figure 2:**
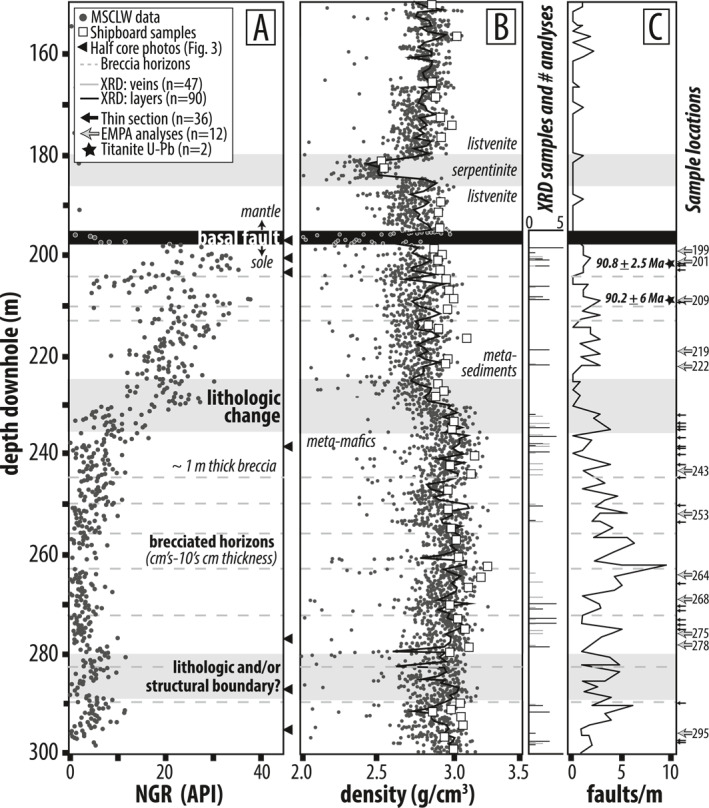
Down‐hole trends in physical properties (data from Kelemen et al., 2020) and structure for core BT‐1B from 150 to 300 m (bottom of the hole is 300 m). Depths and number of XRD analyses, thin sectioned samples, and samples analyzed via EMPA and/or U‐Pb are indicated. Numbers next to sample depths are meters down‐hole. Gray dots are from Multi‐Sensor Core Logging of Whole‐round cores (MSCL‐W) and white squares in (b) are measurements from shipboard samples. Black triangles mark half core photos shown in Figure 3. (a) Natural Gamma Radiation (NGR) in American Petroleum Institute (API) units. (b) Density in g/cm^3^ (c) Down‐hole abundance of brittle faults (mm‐scale to cm‐scale offset, normal and thrust sense) per meter. Dashed gray lines are brecciated horizons, cm to 10's of cm thick.

The Oman Drilling Project (OmanDP) was a multinational research endeavor motivated to better understand the processes that create and modify oceanic crust and shallow mantle lithosphere. OmanDP sampled key components of the Samail Ophiolite from the crust through the “Basal Thrust” in nine diamond‐cored and six rotary‐drilled boreholes. In March 2017 at Site BT‐1B (23°21.861’ N, 58°20.149’ E), on the north side of Wadi Mansah and ∼50 km NW of Wadi Tayin, 300 m of core was extracted with 100% recovery (Figure 2). BT‐1B comprises 196 m of mantle rocks, the “Basal Thrust” fault zone, and 104 m of sub‐ophiolite metamorphic rocks. The depth to the underlying Hawasina continental margin platform sediments and Haybi volcanic rocks is unknown, but probably less than several hundred meters. Herein, we refer to the “Basal Thrust” as the “Basal Fault”, since most recent displacement along this structure is likely extensional and associated with post‐obduction unroofing and exhumation. Site BT‐1B is the only OmanDP core that sampled the Basal Fault and sub‐ophiolite metamorphic sole rocks.

Mantle rocks above the Basal Fault at Site BT‐1B are a globally rare assemblage of listvenite (carbonated peridotite) containing lenses of serpentinite. The key OmanDP objective at Site BT‐1B was to conduct a detailed study of chemical and structural processes controlling mass transfer along the shallow subduction interface into the overriding mantle wedge, which can absorb carbon in the form of Mg‐carbonates (Falk & Kelemen, 2015; Kelemen & Manning, 2015). Characterization of the sub‐ophiolite metamorphic sole provides some insight into the timing of listvenitization relative to juxtaposition of the sole below the ophiolite and the potential sources of CO_2_‐rich fluids linked to low‐temperature listvenitization (∼50–250°C, Beinlich et al., 2020). We summarize these insights in Section 6 for the benefit of the drilling project, but this was not the primary focus of this study.

On‐site core characterization revealed that the sub‐ophiolite metamorphics did not contain the HT garnet‐bearing sole, instead comprising 104 continuous meters of “low‐temperature” (LT) sole. Therefore, in an idealized ophiolite‐sole sequence, these rocks belong to a structurally deeper section than is typically studied (see gray box in Figure 1b); if the HT sole was originally present between the LT sole and the basal peridotites, it has been tectonically removed. Descriptions of comparable outcrop thicknesses of the LT sole are rarely reported despite being apparently quite common. One locality that exposes both HT and LT sections of metamorphic sole in Oman crops out ∼250 km to the NW at Sumeini and comprises ∼80 m of HT sole overlying ∼20–30 m meters of LT sole (Cowan et al., 2014; Searle & Malpas, 1980; Soret et al., 2017). At Asimah, at least ∼70–80 m of faulted garnet‐bearing amphibolite sole is exposed and transitions downward into LT sole rocks (of unknown thickness) composed of epidote + quartz amphibolites and quartzites (Allemann, 1972; Gnos, 1998; Gnos & Peters, 1992; Soret et al., 2017). At Wadi Tayin, the sole consists of ∼230 m of hornblende‐plagioclase amphibolites with only the uppermost few meters containing garnet (Ghent & Stout, 1981; Hacker & Mosenfelder, 1996; Searle & Malpas, 1980; Soret et al., 2017). The distinction between the HT and LT sole at Wadi Tayin is unclear; metasedimentary rocks are found intercalated with metamafic rocks from the base up to ∼10 m from the contact with peridotites. The ≥100 m thick basal section was originally described as a monometamorphic greenschist facies LT sole (Ghent & Stout, 1981), but in fact records epidote‐amphibolite facies metamorphism with a greenschist facies overprint (Hacker & Mosenfelder, 1996).

## 3 Lithologic and Structural Overview

**Figure 3:**
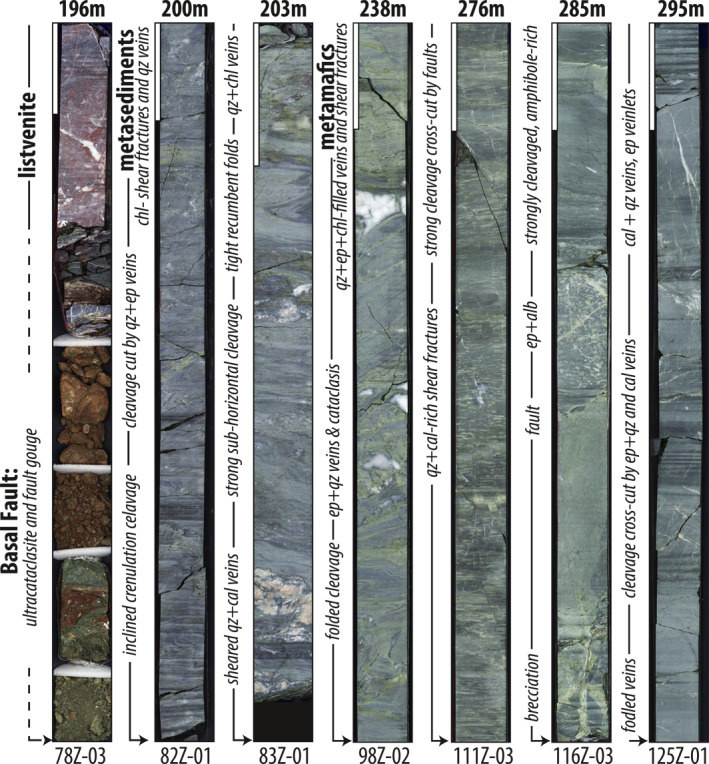
Selected half core photos displaying the structural and lithologic variability in core BT‐1B. At the top of each photo, the starting depth is listed in meters down‐hole. Vertical white bars are 10 cm.

The upper 196 m of core BT‐1B comprise variably deformed, locally mylonitic, white and red (iron oxyhydroxide stained) listvenite and several cm to ∼7 m thick horizons of serpentinite (Figures 2a and 2b). Intervals of breccia cm to 10's of cm thick and cm thick cataclasites are abundant, and planar faults are rare (e.g., Figure 2c; cf. Menzel et al., 2020). At 196 m, the Basal Fault is characterized by a ∼40‐cm‐thick zone of fault gouge and ultracataclasite (Figure 3).

The 104 m section of sub‐ophiolite metamorphic rocks can be subdivided into three sections marked by lithological and/or structural boundaries. The upper 34 m (196–230 m) comprise mostly phyllitic, blue‐gray to gray‐green, metasedimentary rocks (Section 1). Section 1 produces a strong positive excursion in Natural Gamma Ray (NGR) intensity relative to overlying listvenites and underlying meta‐mafics, and the bulk density of the listvenites and phyllites are surprisingly similar at ∼2.7 g/cm^3^ (Figures 2a and 2b; data from Kelemen et al. (2020)). Two collected samples (203 and 209 m) are derived from ∼30 cm‐thick calcite‐bearing intervals, described as calc‐phyllites. A lithologic change marks the transition to Section 2, which comprises ∼60 m (230–290 m) of schistose meta‐mafic rocks containing ∼30%–50% epidote by volume that is visually distinct at the core scale. The transition between Sections 1 and 2 appears gradational in physical properties data (e.g., decrease in NGR and increase in bulk density to ∼2.9 g/cm^3^; Figure 2) and is characterized by a gradual increase of intercalated meta‐mafic material between ∼215 and 230 m, until the core becomes entirely meta‐mafic at ∼230 m. Toward the bottom of Section 2 between ∼275 and 290 m, epidote‐rich schist alternates on the ∼10–50 cm scale with rocks comprising variably deformed, blocky‐to‐elongated epidote + albite aggregates in a fine‐grained amphibole‐rich matrix. Lithologic transitions are typically gradational and therefore may represent some relict, primary compositional layering, but are locally marked by discrete fault boundaries. A lithological and/or structural change marks the transition to Section 3, which comprises 10 m (290–300 m) of fine‐grained, blue‐black, and amphibole‐rich schists. The Section 2 to 3 transition is not as obvious in physical properties data, but may be marked by a small positive excursion in NGR at 290 m (Figure 2a).

Throughout the 104 m of core, penetrative ductile fabrics are cross‐cut by numerous faults with thin cataclastic zones, as well as several zones up to a few tens of cm thick of cataclasis and brecciation (gray dashed lines in Figure 2). The thickest interval logged as breccia in the core occurs at 244 m and is ∼40 cm thick. Planar faults and thin cataclastic breccia zones are more abundant in the meta‐mafics compared to phyllites (Figure 2c). Offset along the thin cataclastic breccia zones is uncertain, but is probably at least tens of centimeters because some fabric discordance is common on either side of brecciated horizons. The amount of offset across most of the planar faults is unknown, but probably small as few juxtapose discernible changes in lithology. The most obvious change in lithology that implies large (probably meters) displacements is present at 285 m where a layer of epidote + albite aggregate‐bearing schist is sharply juxtaposed against a fine‐grained epidote‐amphibole schist (Figure 3, panel 285 m). Planar faults and catalcastic breccias are sparse in the lower 50 m of the carbonated ultramafics.

The dominant foliation is generally near‐perpendicular to the core's long axis and thus is dipping shallowly to moderately, given the Hole BT‐1B inclination of ∼80°. This is consistent with field observations of the listvenite‐sole contact near Site BT‐1B (cf. Menzel et al., 2020). Intervals of core up to ∼70 cm long have a foliation that dips obliquely (up to ∼45°) suggesting that the core may have intersected several meter‐scale folds, such as those reported by Soret et al. (2017).

### 3.1 Mafic Geochemistry and Mineralogic Variations

Four meta‐mafic samples were selected for bulk geochemical analysis. They have immobile trace element signatures characteristic of normal mid‐ocean ridge and alkalic “within‐plate” basalts (Figure S1 and Table S1 in Supporting Information S1). This signature is different than the ophiolite lava chemistry and comparable to compositions reported for other mafic metamorphic rocks of the HT sole (Ishikawa et al., 2005; Searle & Cox, 2002).

To assess the nature of lithologic variations as inferred from color and textural differences, all visually distinct layers were sampled by micro‐drilling for XRD analyses (Dataset S1). The common metasedimentary mineral assemblage (*n* = 22) is dominated by muscovite, quartz, albite, titanite, hematite, and chlorite, with some layers containing detectable epidote, amphibole, apatite, calcite, and potentially stilpnomelane (*n* = 1, but not confirmed with EMPA) (Figure S2 in Supporting Information S1). The common meta‐mafic mineral assemblage (*n* = 68) is epidote, chlorite, albite, and calcic amphibole. Small amounts of muscovite, quartz, and titanite are common and some samples have detectable hematite, apatite, calcite, and potentially stilpnomelane (*n* = 2, but not confirmed with EMPA) (Figure S2 in Supporting Information S1).

### 3.2 Veinlets and Veins

**Figure 4:**
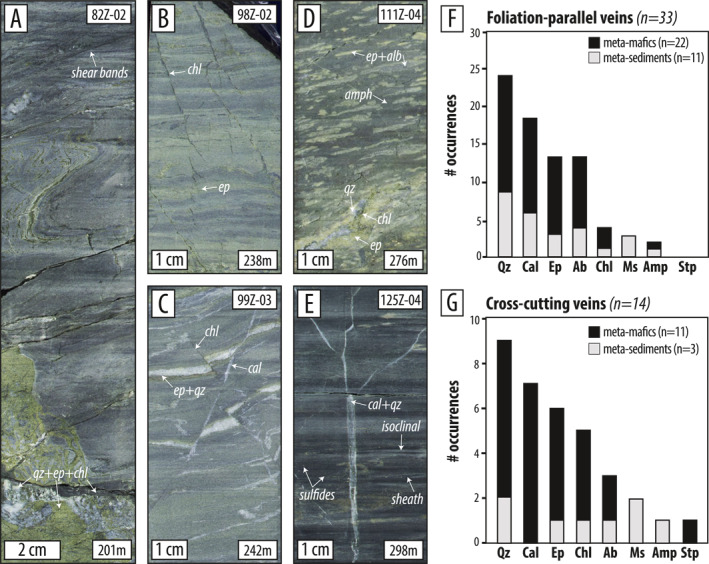
Close‐up half‐core photos showing key structures and cross‐cutting relationships in (a) metasediments (Core Section 1) (b–d) epidote‐rich meta‐mafics (Core Section 2), and (e) amphibole‐rich meta‐mafics (Core Section 3) (f, g) Histograms of XRD results showing foliation‐parallel and cross‐cutting vein mineralogy. See Figure S2 in Supporting Information S1 for more XRD results.

Veinlets (<1 mm thick) and veins (up to ∼1 cm thick) are abundant in the core. While some are cross‐cutting and late in the deformation history, many are clearly folded and some appear to have been transposed. XRD analysis of foliation‐parallel (*n* = 33) and cross‐cutting (*n* = 14) veins reveal quartz, calcite, epidote, albite, and chlorite dominate, usually in bimineralic associations. Older foliation‐parallel and probably transposed veins are dominated by calcite + quartz, quartz + albite, and quartz + epidote (Figure 4f) while younger cross‐cutting veins are more commonly monomineralic quartz or calcite (Figure 4g). Veins in the meta‐mafic rocks are combinations of quartz, calcite, epidote, albite, and chlorite; these mineralogies are common in greenschist facies terranes. Most veins in the meta‐mafic rocks crosscut metamorphic fabrics.

## 4 Deformation and Metamorphism in the Low‐Temperature Sole

Thirty‐six samples were collected from the metamorphic sole in the core and examined petrographically (Figure 2, black and gray arrows in panel C) in order to establish the deformation‐metamorphism history of the LT metamorphic sole in core BT‐1B. Twelve samples, representative of the full range of metamorphic variations, were selected for optical microstructural study and quantitative Electron Microprobe Analyses (EMPA) (5 metasediments and 7 meta‐mafics; gray arrows in Figure 2c). Three deformation and metamorphic stages (*D*_1−3_) were identified and described in terms of their dominant foliation‐ and lineation‐forming mineralogy, mineral stability, and kinematic context (pre‐, syn‐, and post‐kinematic with respect to surrounding fabric), breakdown and replacement textures, and whether metamorphism was dynamic (i.e., metamorphic growth in a preferred orientation) or static (i.e., randomly oriented growth). Samples containing large blue‐green amphibole crystals (≥1 mm in length) were targeted for EMPA analyses at the University of Texas at Austin on the JEOL JXA‐8200 in the Department of Geological Sciences (details in Text S2 and S3 in Supporting Information S1).

**Figure 5:**
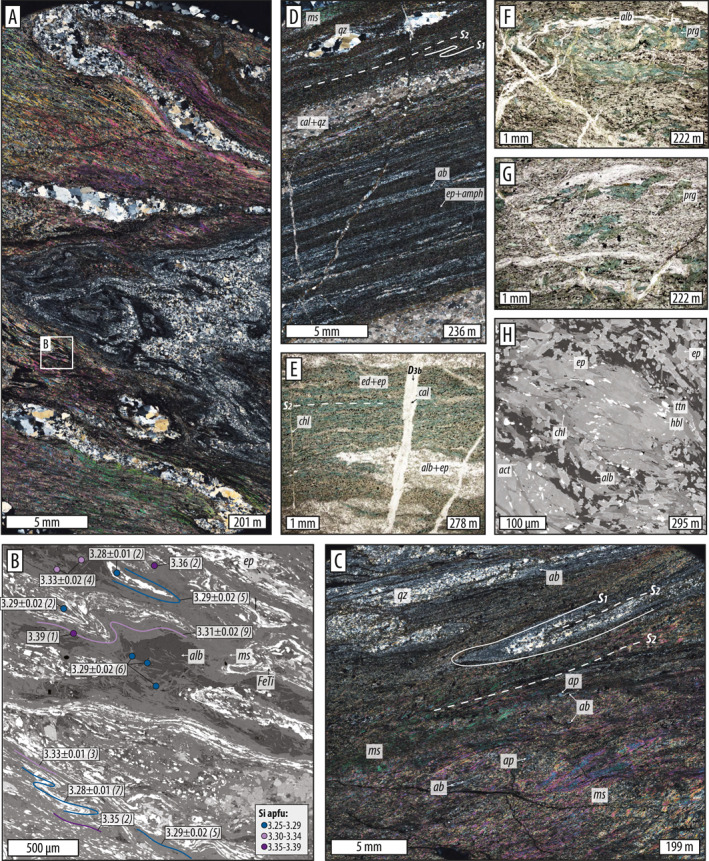
Representative photomicrographs of D_1_ and D_2_ microstructures in metasediments (a–c) and meta‐mafics (d–h). Ductile foliations contain the axial planes of isoclinal folds (a–c, f), fish‐hook and eye structures (a, c), and boudinaged and transposed veins (a, d). (b) BSE Image of area corresponding to white box in (a), with Si‐in‐phengite results shown (e, f) Amphibole‐rich S_2_ foliations in meta‐mafics comprise well‐aligned edenite and pargasite (f, g) Pargasite is stable during D_2_ folding and shearing (h) Syn‐D_2_ amphibole zonations trend from actinolite to magnesio‐hornblende (or edenite/pargasite). Mineral abbreviations: act, actinolite; alb, albite; aln, allanite; cal, calcite; chl, chlorite; ed, edenite; ep, epidote; FeTi, Fe‐Ti oxide; hbl, hornblende; ms, muscovite, prg, pargasite, and ttn, titanite.

**Figure 6:**
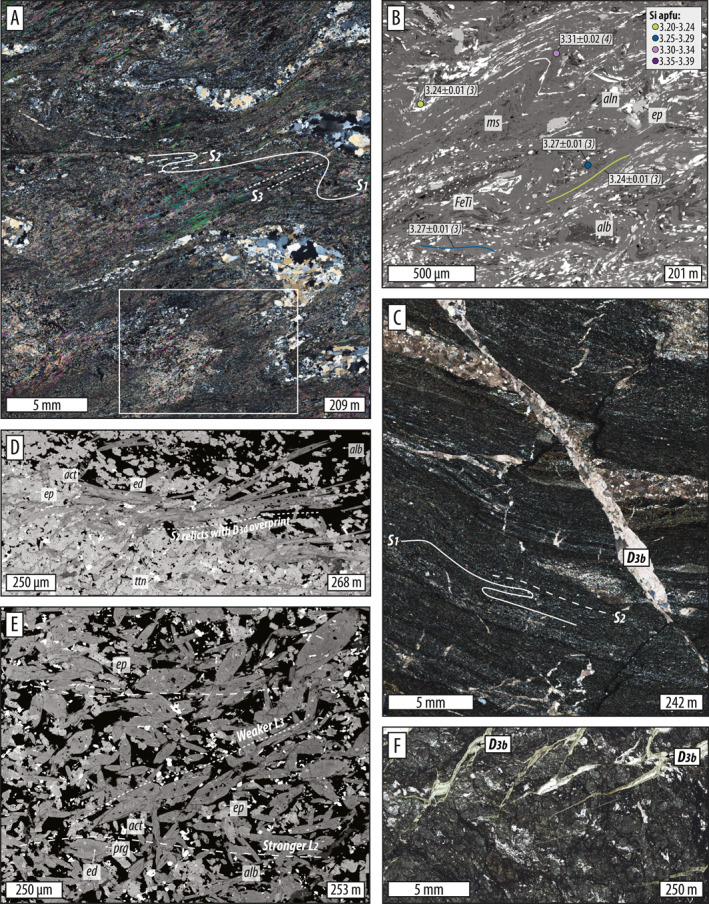
Representative photomicrographs of D_3_ microstructures in metasediments (a, b) and meta‐mafics (c–f). S_1_ and S_2_ foliations are crenulated during D_3_, locally forming a new axial planar cleavage, S_3_ (a, b). (b) BSE image with Si‐in‐phengite results shown. White box in (a) corresponds to false‐colored X‐Ray map in Figure 9. (c) Late cross‐cutting veins contain calcite and record normal sense of shear. (d) D_3_ amphibole zonations (actinolite) overprint the S_2_ foliation. (e) One stronger (S_2_, older) and one weaker (D_3_, younger) lineation defined by different amphibole core‐rim zonations. (f) Chlorite‐filled veins crosscut brecciated meta‐mafic rock. Mineral abbreviations: act, actinolite; alb, albite; aln, allanite; chl, chlorite; ed, edenite; ep, epidote; FeTi, Fe‐Ti oxide; hbl, hornblende; ms, muscovite; prg, pagasite; and ttn, titanite.

The following results support the interpretation that D_1_ and D_2_ record progressive development of prograde penetrative strain (Figure 5). A third, distinct stage of overprinting under lower temperature conditions was identified as deformation stage D_3_ (Figure 6). Dynamic deformation and metamorphism and ductile fabric regeneration during D_1_ and D_2_ became progressively less penetrative during D_3*d*_ (*d* for ductile), and transitioned to a stage of brittle, less pervasive faulting and veining during D_3*b*_ (*b* for brittle).

### 4.1 D_1_ and D_2_ Deformation and Metamorphism

#### 4.1.1 Microstructures

No primary depositional or magmatic textures were observed in the phyllitic or meta‐mafic units. Two stages of penetrative ductile strain are identifiable in most thin sections. In phyllitic metasedimentary rocks, the oldest stage, D_1_, developed a strong mica‐rich foliation, S_1_. This foliation contains mm to 10's of cm scale layers that are rich in quartz + albite ± epidote and/or calcite (Figures 5a–5c). Some foliation‐parallel syn‐D_1_ compositional layering are quartz, quartz ± albite, and quartz ± epidote veins that were transposed parallel to the developing foliation and ductilely pinched (Figure 5a). The S_1_ fabric was folded during D_2_, which is best seen in deformed compositional layers and veins. D_2_ folding varies in geometry from open, to tight, to isoclinal (Figures 5a and 5c) and locally formed an S_2_ axial planar cleavage that is sub‐parallel to S_1_. S_2_ is best developed in mica‐rich layers. Locally, D_2_ folding produced rootless fishhook and eye (sheath) structures, indicating that shear strain was large (Figures 5a–5c). The S_2_ foliation contains syn‐kinematic porphyroblastic apatite (50–500 μm) and epidote with allanite cores (10–50 μm) fringed by albite pressure shadows, and titanite with white mica pressure shadows (Figures 9a–9c).

The schistose meta‐mafic rocks record a similar D_1_ and D_2_ fabric evolution. The dominant foliation comprises alternating, mm to cm thick, epidote‐albite and amphibole‐epidote compositional layers (S_1_, Figures 5d–5h). The S_1_ schistosity contains preferentially aligned blue‐green amphibole, white mica, and chlorite that define a strong lineation (Figures 5e and 5h) and contains fully transposed, syn‐D_1_ quartz, quartz ± albite, and quartz ± calcite veins, some of which exhibit ductile pinch‐and‐swell textures (Figure 5d). S_1_ was folded into open‐to‐tight and isoclinal microfolds during D_2_. D_2_ locally developed an S_2_ axial planar cleavage that is sub‐parallel to S_1_. D_2_ folding is best seen in relatively coarse‐grained amphibole‐rich layers (Figure 5f). Amphibole porphyroblasts grew parallel (syn‐D_1_ and D_2_) and oblique to the folded foliation; oblique blasts are syn‐D_2_ since they have inclusion trails of Fe‐Ti oxides and white mica that are continuous with the external S_2_ foliation (Figure 5g). The S_2_ foliation contains syn‐kinematic porphyroblastic titanite (10–100 μm) and epidote with allanite cores (10–50 μm) (Figure 5h). We did not find rutile in any of these rocks.

#### 4.1.2 Mineral Chemistry

S_1_‐ and S_2_‐forming white mica in metasedimentary rocks falls on a solid solution between muscovite and celadonite. K atoms per formula unit (apfu) are between 0.8 and 1.0 and paragonite component is ≤0.04 (Figure S3 in Supporting Information S1). Si apfu are elevated with respect to ideal muscovite and span ∼3.1–3.3, approaching “ideal phengite” (Figure S3 in Supporting Information S1). There is no clear trend in Si apfu with depth in the core, but the deepest sample (222) is an intercalated mafic lens within the dominantly metasedimentary section and contains mica with the highest measured Si apfu (∼3.3–3.6) (note that this sample has a different bulk chemistry than the others). EDS analyses confirm that S_1_ and S_2_ plagioclase is albite, with calcium content below detection.

White micas in metasedimentary rocks appear to record differences in Si content corresponding to micro‐structural context. The oldest foliation, S_1_, is characterized by Si apfu of ∼3.2–3.3 (Figure 5b; S3B). Partially recrystallized micas defining the S_2_ cleavage record slightly higher Si apfu reaching ∼3.35–3.40, but tend to overlap with S_1_ phengites within error (Figure 5b; S3B).

**Figure 7:**
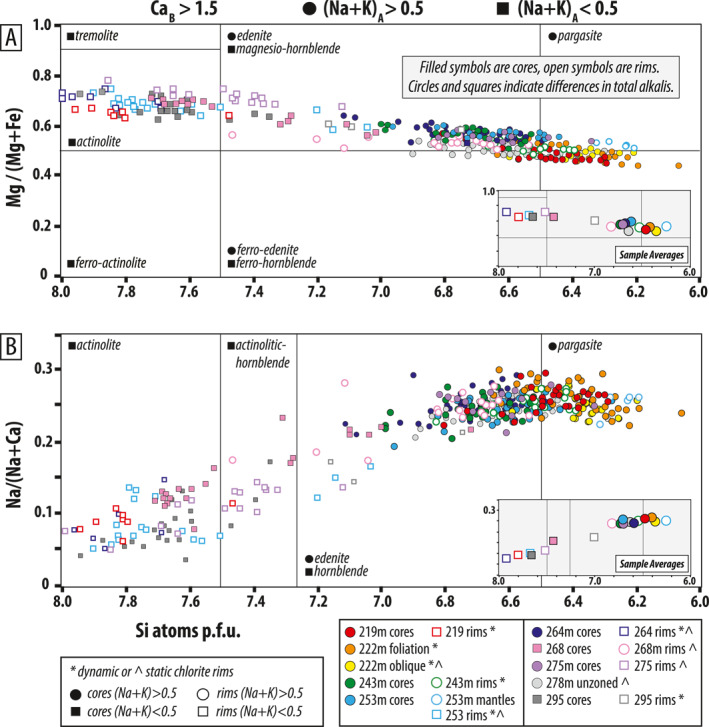
EMPA amphibole analyses. Filled symbols are cores, open symbols are rims. Circles indicate (Na + K)_*A*_ > 0.5, squares indicate (Na + K)_*A*_ < 0.5. (a) Si atoms p.f.u. vs. Mg#. (b) Si atoms p.f.u. vs. Na/(Na + Ca). Fields are labeled according to Leake et al. (1997) classification. Data are colored by depth down‐hole, with warmer colors corresponding to shallower depths. Sample averages for cores and rims are shown in the gray inset boxes.

Amphiboles defining the S_1_ and S_2_ foliations in meta‐mafic rocks record core‐to‐rim zonations characterized by decreasing Mg#, decreasing Si apfu, and increasing Na/(Na + Ca) (e.g., Figure 5h). This zoning was documented in samples 243, 253, 268, and 295 (Figures 7a and 7b) and captures a change from actinolite or actinolitic‐hornblende to magnesio‐hornblende or edenite. Sample 243 is an exception; it has hornblende cores and pargasite rims (Figure 7). Zonations are thin or absent on the tops of elongated crystals, indicating enhanced dissolution perpendicular to foliation, and are thicker parallel to lineation (Figure 5h). No zoning was found in samples 222 and 278; the homogeneous grains of S_1_‐ and S_2_‐forming amphibole are (ferro‐) pargasite and (ferro‐) edenite, respectively (Figures 5e–5g and 7). In sample 222, S_1_‐defining blue‐green pargasite is stable during D_2_ folding, and S_2_ contains syn‐kinematic unzoned pargasite (Figure 5f).

**Figure 8:**
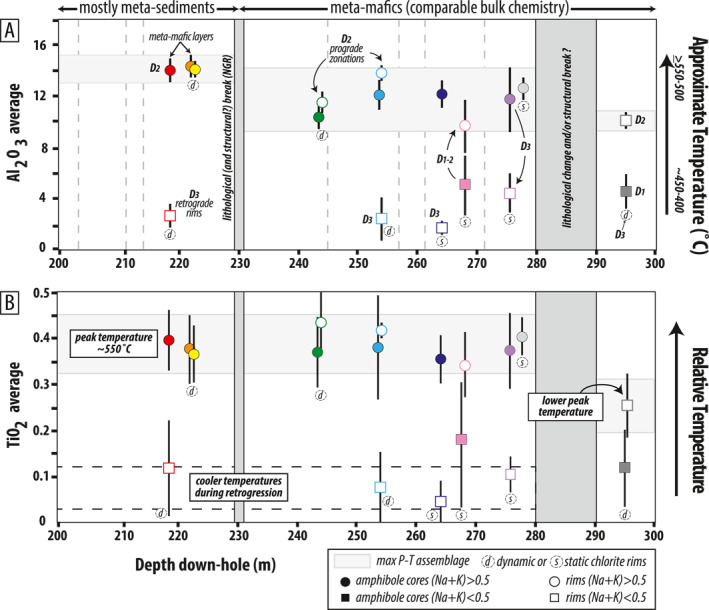
Downhole trends in amphibole chemistry. Vertical dashed gray lines in (a) are brecciated horizons. Samples with dynamic and static chlorite rims are indicated. (a) Maximum Al_2_O_3_ and (b) TiO_2_ in syn‐D_2_ amphiboles decrease slightly with depth. Actinolite overgrowths record D_3_ retrogression. Approximate temperatures based on Al‐ and Ti‐contents are from Ernst and Liu (1998).

Maximum Al_2_O_3_ in S_2_‐defining amphiboles decreases with depth in the core, from ∼13–15 wt% between ∼196–230 m, to ∼10–13 wt% between ∼230–300 m (Figure 8a). Maximum Al_2_O_3_ in sample 295 is also ∼10 wt%, but these rims are distinct from other samples because they have (Na + K)_*A*_ <0.5 (similar to 268 cores). Maximum TiO_2_ drops markedly across the ∼280–290 m horizon from ∼0.4 wt% to ∼0.25 wt% (Figure 8b).

### 4.2 D_3_ Deformation and Metamorphism

#### 4.2.1 Microstructures

**Figure 9:**
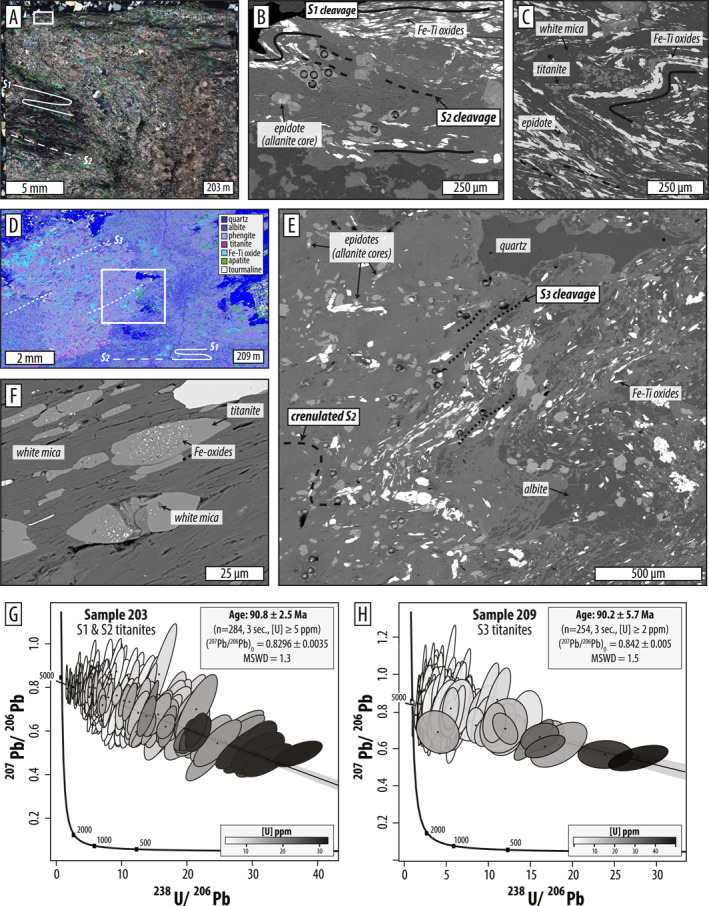
Microstructures of titanite‐bearing rocks selected for U‐Pb geochronology. (a–c) Calc‐phyllite sample AK203. (b) BSE image showing examples of syn‐kinematic S_1_‐S_2_ titanites (laser pits show some analyzed grains). (d) False‐colored X‐Ray map of area in white box in Figure 6a of calc‐phyllite sample AK209. (e) BSE image of white box in (d) showing pre‐to‐syn‐kinematic S_3_ titanites (laser pits show some analyzed grains). (f) Titanites vary from pure and inclusion‐free (more common in sample 203) to inclusion‐rich (more common in sample 209); inclusions are Fe‐oxides and white mica (g, h). Regressions of titanite U‐Pb data plotted in Tera‐Wasserburg space. Total number of data points, *n*, is the total number of 3‐s integrations with [U] ≥ 5 and 2 ppm, respectively.

In metasedimentary rocks in the upper 34 m of the core, the S_2_ foliation was refolded producing D_3_ crenulations (Figures 6a and 6b). Locally, mica‐rich layers developed strong S_3_ cleavages that are parasitic to axial planes of larger, cm‐scale folds visible in thin section (Figure 6a). D_3_ crenulations contain pre‐to‐syn‐kinematic titanite porphyroblasts (∼30–300 μm) that have rotated into alignment with the S_3_ cleavage (Figures 9d and 9e), suggesting that crystals continued growing during D_3_.

D_3_ crenulations do not manifest clearly in meta‐mafic rocks. The best evidence for folding is several changes in apparent dip direction of the meta‐mafic S_2_ foliation with depth down‐core. In most samples, amphibole porphyroblasts define one strong and one weak lineation, potentially recording the older S_2_ and a younger, weak S_3_ fabric, respectively. The younger, weaker fabric is reported herein as D_3*d*_ (*d* for ductile) (Figure 6e). Syn‐kinematic amphibole zonations support the inference that deformation continued under P‐T conditions different than D_2_ (chemistry discussed below). Where the S_2_ foliation records the best evidence of D_3_ overprint, it occurs as lineation‐parallel green amphibole and dynamic chlorite rims replacing amphibole. D_3_ chlorite also grew in brittle micro‐boudin necks dissecting amphibole porphyroblasts, and formed patchy replacement textures and static pseudomorphs of what might have been garnet in one sample (no pristine garnet was found).

During D_3_ in all rock types, S_2_ was locally crosscut by veins, micro‐faults, thin zones of cataclasis, and brecciation (i.e., D_3*b*_, *b* for brittle) (Figures 4, 5, 6). Millimeter‐to cm‐scale thrust‐sense chloritized micro‐faults occur through much of the meta‐mafic section (e.g., Figures 4b and 4c) and are crosscut and offset by normal‐sense, calcite‐filled micro‐faults and veinlets (Figures 4c, 4e and 6c).

#### 4.2.2 Mineral Chemistry

White micas defining D_3_ fabrics trend toward lower Si contents than D_1_ and D_2_ fabrics. Where S_1−2_ cleavages are preserved in crenulation hinges, they preserve higher Si contents (≥3.3 apfu), but where the D_3_ crenulation forms a strong S_3_ cleavage, Si contents are locally reduced to 3.10–3.3.25 apfu (Figures 6b and 6S3b).

Amphiboles that define the syn‐D_3*d*_ overprint of the S_2_ foliation record core‐to‐rim zonations characterized by increasing Mg#, increasing Si apfu, and decreasing Na/(Na + Ca) (e.g., Figures 6d and 6e). This zoning was documented in samples 219, 264, and 275 (Figures 7a and 7b) and corresponds to a change from pargasite or edenite to actinolite. Similar to D_1−2_ zonations, D_3*d*_ amphibole zonations developed as the fabric was continuously reworked. Thicker metamorphic rims grew in the lineation direction (Figures 6d and 6e). Sample 253 is unique in that both kinds of zoning are present; edenite cores evolve to lower Mg#, higher Na‐content pargasite mantles, and then to higher Mg#, higher Si, and lower Na‐content actinolite rims (Figures 6e and 7). Actinolite is commonly in textural equilibrium with foliation‐parallel D_3*d*_ chlorite, but in several samples chlorite occurs only as patchy replacement textures and static overgrowth (asterisks vs. carets in the key in Figure 7). D_3*d*_ actinolite rims have consistently low Al_2_O_3_ and TiO_2_ of ∼2–4 wt% and ∼0.05–0.1 wt%, respectively, regardless of sample depth (Figures 8a and 8b).

## 5 LA‐ICP‐MS Titanite U‐Pb Geochronology

LA‐ICP‐MS titanite U‐Pb data were collected from the two metasedimentary calc‐phyllite samples that contained the largest and most abundant titanite in order to constrain the timing of progressive fabric development in the LT sole in core BT‐1B. Grains were analyzed in‐situ on ∼30‐μm‐thin sections in order to retain the microtextural context of porphyroblasts, which allowed us to interpret ages in the context of progressive deformation (Figure 9, S5). Careful BSE imaging and EDS spot analysis of each dated grain was employed to ensure that regressed spot‐data were from titanite. Analyzed allanite or intergrown titanite grains were removed from any data regression.

Samples were analyzed at the University of Texas at Austin UTChron Laboratory using a 193 nm ArF Excimer laser ablation system with a Helex cell coupled to an Element 2 HR‐ICP‐MS. Titanites were ablated using 30 μm spot sizes for 30 s at 10 Hz with an energy of 4 mJ. OTL‐1 was used as primary titanite standard (1,015 ± 2 Ma; Kennedy et al., 2010) and interspersed every 4 unknown analyses for elemental and depth‐dependent fractionation. BLR‐1 (1,047 ± 0.4 Ma; Aleinikoff et al., 2002; Bonamici et al., 2015) was used as a secondary standard for quality control. No ^204^Pb‐based common Pb correction was applied due to the interference with ^204^Hg in the He and Ar gases. Data reduction was performed using the IgorPro (Paton et al., 2010) based Iolite 3.4 software with a Visual Age data reduction scheme (Petrus & Kamber, 2012). All bulk and 3‐s data are given at 2‐sigma with internal and external uncertainty propagated. Data were regressed and lower intercept ^206^Pb/^238^U ages were calculated using IsoplotR (Vermeesch, 2018). All data are presented in Data Set S2.

In light of the low U and variable Pb_*c*_ content, LA‐ICP‐MS U‐Pb analyses of titanite may be hampered and the precision limited, as they do not yield concordant ages. The variable Pb_*c*_ content or [U], however, can be leveraged in Tera‐Wasserburg space to define inverse isochrons (i.e., mixing trajectories between radiogenic and common Pb). While ages can be calculated assuming a Stacey and Kramers (1975) model Pb composition, it is far more strategic to leverage internal titanite U and Pb variability to define more robust regressions and deriving lower‐intercept ages without making model Pb composition assumptions. Traditionally, studies use different bulk titanite spot analyses to regress intercept ages and Pb_*c*_ composition, but for this study, we utilized an approach similar to depth profiling (cf. Odlum & Stockli, 2019) to resolve intra‐grain U and Pb_*c*_ variations and to refine lower intercept age precision. Rather than analyzing time‐ and/or depth‐dependent differences in core vs. rim ages and chemistry (as is common for traditional depth profiling), we analyzed individual titanite grains in thin section—thus sampling some cross‐sectional area through potentially zoned single crystals—with relatively large laser footprints comparable to the diameter of individual titanite grains. We then subdivided the continuous 30 s ablation trace into 3‐s (∼2 μm) segments to capture the variability in U and Pb_*c*_ in sub‐domains of individual grains and regressed these 3‐s U‐Pb analyses. This technique allows more precise regression of data with low [U]. See Text S4, Table S5 in Supporting Information S1 and Figures S6 and S7 in Supporting Information S1 for more information on U‐Pb analyses and age calculations.

Sample AK‐82Z‐03‐202.9 (IGSN: IEBTB000M) is a quartz‐mica coarsely phyllitic calcareous layer that displays a strong D_2_ fabric (Figures 9a–9c). The S_2_ cleavage is well‐developed in white mica and Fe‐Ti oxide‐rich lenses and is axial planar to cm‐scale folds (Figure 9a). Subsequent warping of the D_2_ foliation is evident in small crenulations that kinked mica and locally rotated titanite (see Figure S5 in Supporting Information S1) but this deformation was not penetrative. Titanite blasts (∼10–200 μm) are pre‐to‐syn‐D_2_ as evidenced from their fish‐shaped geometries and alignment with S_2_ axial planes (Figures 9b and 9c). Titanites in this sample are mostly inclusion‐free and are not zoned in Z‐contrast images (Figure S5 in Supporting Information S1). Overall, 83 titanite grains were analyzed and the data regressed as 3‐s increments and filtered for [U] ≥ 5 ppm. Of these data, only grains (*n* = 53) that were confidently identified as syn‐D_1_ and syn‐D_2_ titanites were used in the final regression. BSE imaging and EDS analysis identified 34 allanite grains that were analyzed in the original sequence; these data were removed from the regression. In Tera‐Wasserburg space, 284 datapoints yielded a lower‐intercept age of 90.8 ± 2.5 Ma (MSWD = 1.3; Figure 9g). For comparison, bulk regressed data from 53 S_1_ and S_2_ titanites yielded a lower‐intercept age of 91.8 ± 3 Ma (MSWD = 1.6). We consider the 3‐s integrated data a more robust result due to greater spread in [U] and a slightly better MSWD.

Sample AK‐86Z‐04‐209.35 (IGSN: IEBTB000O) is a quartz‐calcite‐mica phyllite that records evidence for D_3*d*_ (Figures 6a, 9d–f). The strong S_2_ isoclinally folded foliation was refolded into D_3*d*_ folds, producing an axial planar S_3_ cleavage in white mica and albite‐rich layering (Figure 9d). Fe‐Ti oxides, apatite and titanite porphyroblasts, and quartz‐rich microlithons define D_3*d*_ microfolds. Most titanite blasts (∼30–150 μm) are rotated into alignment with the S_3_ cleavage (Figure 9e). In BSE images, titanites appear to lack Z‐contrast zonations and occur both as inclusion‐free grains and grains with inclusion‐rich cores and inclusion‐free rims. The most common inclusions are Fe‐oxides and white mica; no zircon inclusions were identified (Figure 9f). A total of 75 titanite grains were analyzed and the data were regressed in 3‐s increments and filtered for [U] ≥ 2 ppm. Of these data, only grains (*n* = 43) that were confidently identified as syn‐D_3_ titanites were used in the final regression. BSE imaging and EDS spectra identified 2 allanite grains that were analyzed in the original sequence; these data were removed from the regression. In Tera‐Wasserburg space, 254 datapoints yielded lower‐intercept age of 90.2 ± 5.7 Ma (MSWD = 1.5; Figure 9h). The lower precision compared to sample 203 is partly related to lower U content (hence the less restrictive [U] filter). Bulk regressed data from 45 S_3_ titanites yielded a much less precise lower‐intercept age of 123 ± 13 Ma (MSWD = 1.9). We consider the 3‐s integrated data a more robust result due to greater spread in [U], a better MSWD, and that crosscutting relationships indicate the S_3_ fabric must be younger than the S_1−2_ fabric.

## 6 Interpreted Tectonic Context, P‐T Conditions, and Timing of Deformation and Metamorphism in the LT Sole at Site BT‐1B

Since there are no significant differences in major element chemistry between the four meta‐mafic samples that were analyzed (cf. Table S1 in Supporting Information S1), we interpret the differences in amphibole composition to reflect changes in P and T during deformation. This interpretation is supported by Al‐in‐hornblende barometry and amphibole‐plagioclase thermometry multi‐equilibrium calculations presented below. Differences in Si‐in‐phengite are, however, primarily a reflection of different bulk compositions (e.g., sedimentary vs. mafic protoliths, discussed below). Due to complexities with estimating effective bulk composition during growth of zoned amphibole and poorly constrained amphibole solution models (e.g., Diener et al., 2007; Diener & Powell, 2010; Forshaw et al., 2019), we do not present forward thermodynamic modeling (i.e., pseudosections) here, but this will be the focus of future work. Multi‐equilibrium thermobarometry presented herein does not rely on estimating bulk rock composition or activity‐composition models.

### 6.1 Evolving Conditions and Timing of Prograde Metamorphism

**Figure 10:**
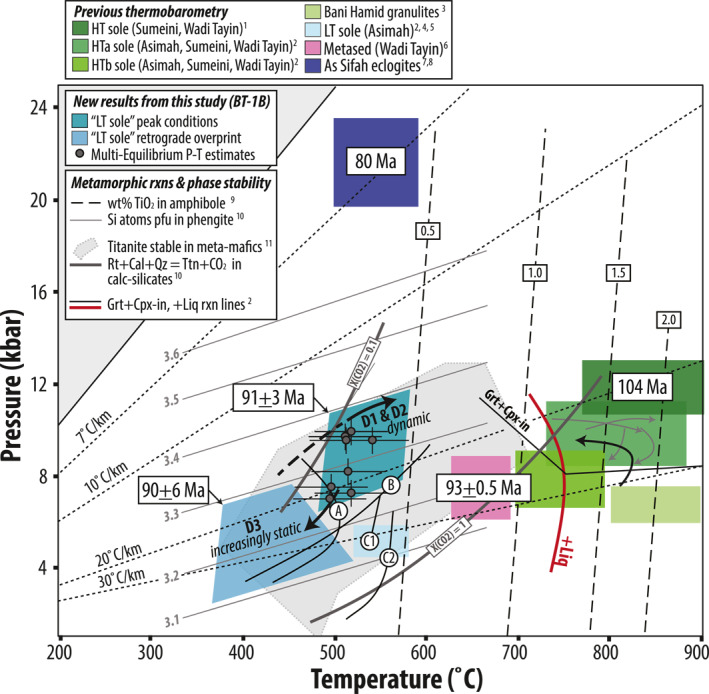
Estimated P‐T conditions and timing of metamorphism in the LT sole in core BT‐1B (teal colors), compared with the HT sole (green and pink) and As Sifah eclogites (dark blue) in NE Oman. Semi‐quantitative peak P‐T estimates (gray datapoints) were derived from Al‐in‐amphibole barometry (Schmidt, 1992) and amphibole‐plagioclase thermometry (Holland & Blundy, 1994), using the iterative approach of Anderson and Smith (1995); see also Figure 11. The gray P‐T trajectories for the HT sole are from Soret et al. (2017); black counterclockwise trajectory is from Hacker (1991). References: (1) Cowan et al. (2014), (2) Soret et al. (2017), (3) Searle et al. (2015), (4) Gnos (1998), (5) Bucher (1991), (6) Garber et al. (2020), (7) Wendt et al. (1993), (8) Searle et al. (1994). Contours for wt% TiO_2_ in amphibole from (9) Ernst and Liu (1998). Contours for Si atoms p.f.u. in white mica from (10) Massonne and Schreyer (1987). Titanite stability in meta‐mafic rocks and calc‐silicates (for end‐member fluid compositions) from (11) Kohn (2017). Metamorphic reactions: (a) actinolite‐hornblende and (b) albite‐oligoclase transitions after Maruyama et al. (1983); (c) Chl + Ttn + Qz + Act = Hbl + Ilm + fluid, buffered by (1) nickel‐nickel‐oxide and (2) quartz‐magnetite‐fayalite after Liou et al. (1974). Ages of metamorphism are from Guilmette et al. (2018) (HT sole), Garber et al. (2020) (structurally highest metasediment, 12 m beneath peridotites at Wadi Tayin), this study (LT sole), and Warren et al. (2005) (As Sifah).

During D_1−2_, S_1,_ and S_2_ developed as P and T increased during subduction. Phengitic white mica appears to record a slight increase in Si apfu during S_2_ cleavage development, indicative of increasing pressure conditions (Figure 10). Experimental calibration of the phengite geobarometer was performed with coexisting K‐feldspar, quartz, and phlogopite (Massonne & Schreyer, 1987; Massonne & Szpurka, 1997; Velde, 1965). Both the metasedimentary and meta‐mafic samples in this study lack this buffering assemblage, but the experimental calibration can still be used to make qualitative assessments of relative pressure changes, and to estimate minimum pressures if the white mica crystallized in the presence of a Mg‐Fe silicate (cf. Massonne & Schreyer, 1987). In meta‐mafic sample 222, Mg‐Fe amphibole is a foliation‐forming phase, and S_2_‐defining phengite records ∼3.5 Si apfu, suggesting D_2_ occurred at minimum pressures of ∼8–12 kbar for temperatures of ∼400–600°C (Figure 10). The other phengite data (samples 199, 201, and 209) come from metasedimentary phyllites that contain neither a buffering mineral assemblage nor a coexisting Mg‐Fe silicate. In those samples, S_1_ and S_2_ schistosities record a tight data cluster of ∼3.1–3.3 apfu. Since there are no obvious structural discontinuities within the 34‐m‐thick metasedimentary section, it appears this portion of the core, while highly sheared, reached roughly the same depths of burial at the culmination of D_2_.

S_1_ and S_2_‐defining amphibole evolved from actinolite to magnesio‐hornblende/edenite, or edenite to pargasite. The exact core‐to‐rim evolution and type of amphibole that grew was likely a function of local differences in reactive bulk composition and strain. Rare actinolite cores, observed in 2 of 9 meta‐mafic samples analyzed, preserve evidence for prograde subduction through greenschist facies conditions (Figure 10; 268 and 295 m), but most samples appear to have fully equilibrated at lower‐amphibolite facies conditions. Combined with other petrologic constraints, maximum values of Al_2_O_3_, TiO_2_, and (Na + K) apfu in syn‐D_2_ amphibole zones are generally consistent with prograde‐to‐peak metamorphism at ∼8–10 kbar and ∼400–550°C; Al_2_O_3_ values alone could indicate P>15 kbar (Figures 8 and 10 and Figure S4 in Supporting Information S1) (Blundy & Holland, 1990; Ernst & Liu, 1998; Holland & Blundy, 1994; Holland & Richardson, 1979; Laird & Albee, 1981). At the greenschist‐to‐amphibolite facies transition, Al_2_O_3_ in Ca‐amphiboles (i.e., Ca_*B*_ > 1.5, present in all samples analyzed) increases from ∼5–15 wt% (Apted & Liou, 1983; Ernst, 1988), TiO_2_ surpasses ∼0.5 wt% (Ernst, 1988), and Mg/(Mg + Fe) and total Ti, Na, and K contents increase while Si decreases (cf. Figure 7) (e.g., Holland & Richardson, 1979; Laird & Albee, 1981; Raase, 1974). Furthermore, at intermediate pressures of 5–8 kbar, prograde greenschist‐to‐amphibolite metamorphic reactions (*zoisite + chlorite + albite* → *Al‐amphibole + anorthite + H_2_O*) will produce Al‐amphibole faster than it consumes zoisite to produce anorthite, thus giving rise to the epidote‐amphibolite facies characterized by the assemblage hornblende + albite + epidote (Apted & Liou, 1983; Evans, 1990; Maruyama et al., 1983). Combined with the Al‐contents in peak D_2_ amphibole, pressures of 5–8 kbar appear to be a minimum. Furthermore, the lack of jadeite + quartz appear to bracket the upper bounds of the peak pressures to ≤10–12 kbar (for 350–550°C), and lack of rutile indicates P<15 kbar. Maximum *T* of ∼550°C is well‐constrained by amphibole Ti‐contents and is consistent with Raman spectroscopy on carbonaceous material (Figure 10) (Soret et al., 2017). The absence of oligoclase, which replaces albite above the peristerite gap, also indicates T<600°C.

Ambrose et al. (2021) documented similar hornblende and pargasite amphibole compositions to those reported in this study, in epidote‐amphibolite facies sole rocks present ∼85–250 m below peridotites in the Masafi (Asimah) Window (UAE). Their average‐P calculations were restricted to one independent reaction due to limited equilibria, but yielded similar P as those inferred here: ∼9–12 kbar. Our core and rim amphibole analyses shows that most samples record the retrograde D_3_ progression to actinolite composition; exceptions are samples 268 and 278. In sample 268, cores are actinolite and rims are edenite. In sample 278, no zonations were observed with amphibole being homogeneous edenite. Both samples appear to record prograde fabrics, with only local late static chloritization.

We corroborated our peak P‐T estimates with semi‐quantitative multi‐equilibrium thermobarometry. We calculated P‐T estimates for 10 samples that record syn‐D_2_ amphiboles using the Schmidt (1992) Al‐in‐hornblende barometer and the Holland and Blundy (1994) amphibole‐plagioclase thermometer for the calibration reaction *edenite + albite* → *richterite + anorthite*. Amphibole compositions used in the calculations are averages of 3–58 measurements (see Table S4 in Supporting Information S1). We used a plagioclase composition of An3, representing the maximum anorthite component that is typical in low‐grade plagioclase before increasing to An18 across the peristerite gap (Maruyama et al., 1982). We did not quantify plagioclase chemistry with EMPA analyses, so reported uncertainties are minimums. However, this assumption is supported by EDS spectra and XRD analyses. EDS shows most contain pure albite and Ca is undetectable. We acknowledge the lack of Ca could be a result of retrogression, but cannot resolve this further with the present data set.

**Figure 11:**
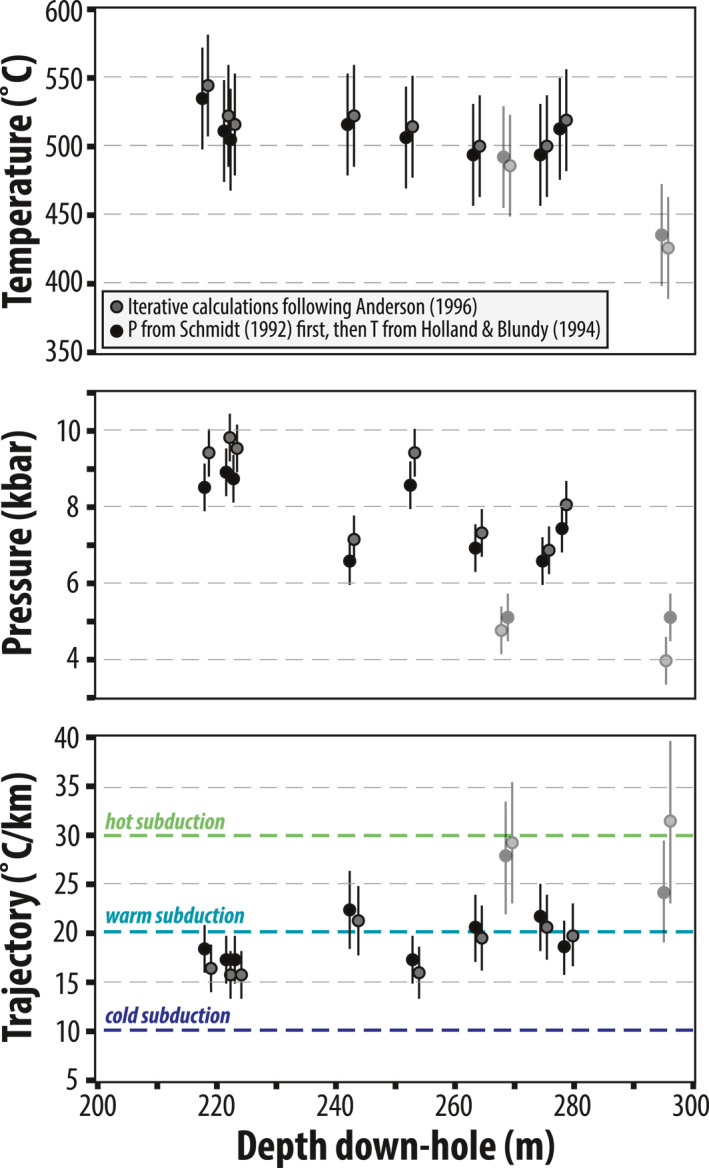
Results from semi‐quantitative multi‐equilibrium P‐T calculations for the LT sole, plotted by depth down‐hole. Gray symbols are preferred results, calculated from the iterative approach of Anderson (1996). Black symbols are results from first calculating pressures from the Al‐in‐hornblende barometer (Schmidt, 1992) and then calculating temperatures from the amphibole‐plagioclase thermometer (Holland & Blundy, 1994). Uncertainties in P and T are from calibration error (±0.6 kbar and 40°C) and do not reflect variations in amphibole chemistry for a given sample or microstructural domain (for which uncertainties would be lower). Plagioclase compositions used for calculations are assumed from EDS spectra and have not been quantified; therefore, uncertainties in temperature estimates are minimums. Uncertainties in P‐T trajectory estimates reflect the range of P‐T from the errors shown in the top two panels. Two samples returned lower P‐T from the iterative approach and much lower P estimates; they are shown in faded symbols.

Two approaches yielded reasonable P‐T estimates that overlap within error and are consistent with the independent petrologic indicators discussed above. The first constrains P from the Al‐in‐hornblende barometer, then calculates T from amphibole‐plagioclase compositions; the second iteratively calculates P and T and accounts for the effects of oxygen fugacity on the Al‐in‐hornblende barometer (Anderson, 1996). Results are shown in Figure 11 and Table S4 in Supporting Information S1. Reported uncertainties are from calibration error (±0.6 kbar and ±40°C) and do not reflect variation in measured amphibole chemistry from individual samples or microstructural domains, which would yield lower uncertainties. The preferred method (see Anderson, 1996) yields peak P‐T for D_2_ spanning ∼6.8–9.7 kbar and 490–540°C. Two samples yielded slightly lower pressures of 4–5 kbar. Down‐core trends in peak P‐T appear to record a weak inverted gradient. However, 8 of the 10 samples record very similar depth‐temperature trajectories of ∼15–22°C/km despite slight differences in peak P‐T (Figure 11).

**Figure 12:**
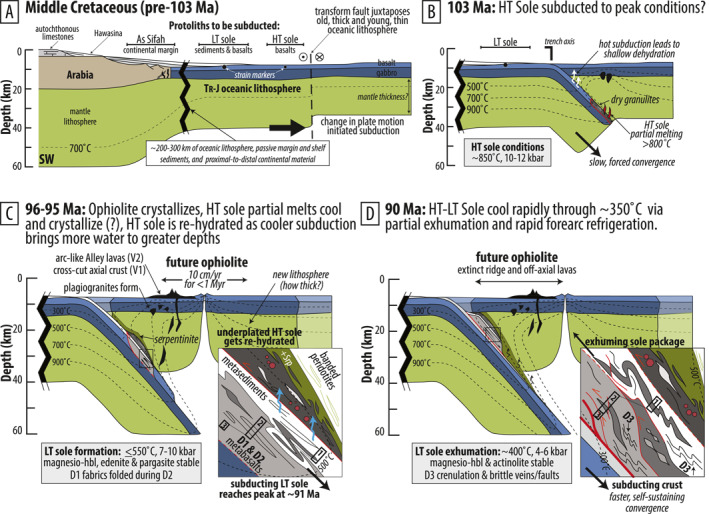
Schematic tectonic reconstruction illustrating a conceptual model for the formation of the metamorphic sole. Compare time stamps with events highlighted in Figure 13. (a) Pre‐subduction configuration. Black circles outlined in white are schematic strain markers. (b) The uppermost HT sole reaches peak conditions at ∼103 Ma (?). Incipient supra‐subduction zone (SSZ) spreading created forearc basalt (FAB) or mid‐ocean ridge‐like basalt (moist‐MORB) geochemical signatures. (c) The LT sole will reach similar peak depths as the HT sole, but under a cooler depth‐temperature trajectory. Cooler subduction feeds more water into the mantle, producing a geochemical subduction signature in ophiolite lavas. Peak conditions will be achieved at ∼91 Ma, after ophiolite formation between 96 and 95 Ma. Inset shows isoclinal folding characteristic of the metamorphic sole, the ductile welding of the HT sole to the overriding ophiolite mantle, re‐hydration of the HT sole during progressive subduction, and the tectonic juxtaposition of the underplated HT‐LT sole units. Black boxes labeled 1–3 and connected by dashed lines correspond to Core Sections 1–3 as observed in the BT‐1B core. During subduction, each section might have experienced slightly different peak P‐T indicative of different depths along the dipping interface, as inferred from the weak inverted metamorphic gradient with depth in the core. (d) The complete HT‐LT sole package, constructed by progressive subduction, tectonic “slicing” and underplating, exhumed via return flow. Ductile shearing will eventually juxtapose Core Section 1 with Sections 2 and 3 during exhumation. Strain localized toward the bottom of the package in the LT sole under greenschist facies ductile‐to‐brittle conditions.

Bucher (1991) and Hacker and Mosenfelder (1996) arrived at similar peak temperature estimates for the LT sole rocks at Wadi Tayin and Asimah; however, their peak pressures were lower (e.g., ∼5 kbar). These studies documented deformation under epidote‐amphibolite facies metamorphic conditions (M1) followed by a greenschist facies overprint (M2). Bucher (1991) reported M1 magnesio‐hornblende, characterized by average ∼0.03 Ti apfu, ∼7.01 apfu Si, and Mg# of 0.42–0.68. Comparable (D_2_) amphiboles reported here, however, are pargasite/edenite and are characterized by ∼0.4–0.5 Ti apfu, ∼6.38–6.58 Si apfu, and Mg# ∼0.4–0.58 (see Table S3 in Supporting Information S1), indicative of slightly higher peak P‐T. It is possible that Bucher (1991) missed the pargasitic amphiboles because they are commonly only preserved in interiors of sub‐mm sized, zoned amphibole crystals. Furthermore, M1 textures were strongly overprinted during dynamic M2 metamorphism under greenschist facies conditions, which erased much of the evidence for the earlier higher‐P deformation phase (Figures 10 and 12).

Abundant titanite and lack of rutile in D_1−2_ fabrics confirm deformation, and metamorphism occurred under moderate P‐T conditions in the presence of H_2_O‐rich, low‐CO_2_ fluids (Figure 10; Kohn, 2017). In light of titanite's relatively high U‐Pb closure temperature (>600°C; Bonamici et al. (2015); Cherniak (1993); Holder and Hacker (2019); Spencer et al. (2013)), its proclivity to participate in metamorphic reactions over a wide range of P‐T space (Frost et al., 2001; Kohn, 2017; Yakymchuk et al., 2017), and our new petrological constraints confirming peak temperatures in the LT sole of <550°C, we interpret the syn‐D_2_ titanite U‐Pb age from sample 203 to record the timing of titanite crystallization and S_2_ cleavage development at ∼91 Ma under epidote‐amphibolite facies conditions. We emphasize that our calculated U‐Pb dates are volumetrically averaged ages of titanite crystallization; however, since titanites in sample 203 are mostly unzoned and inclusion free, we consider this a maximum age of D_2_ deformation in this rock.

### 6.2 Conditions and Timing of Exhumation

During D_3_, deformation and metamorphism continued as P and T decreased from epidote‐amphibolite to greenschist facies conditions. Phengitic white mica recrystallized along S_3_ cleavages; lower Si apfu values in S_3_ cleavage‐defining micas relative to low‐strain relicts of older fabrics preserved in micro‐fold hinges suggest lower pressures during D_3_. D_3_ retrogression overprinted S_2_ and manifested as foliation‐ and lineation‐parallel actinolite rims fringing pargasite or edenite cores and growth of foliation‐forming chlorite, reflecting hydration reactions during cooling and decompression under greenschist facies conditions.

Metamorphic conditions of the ductile D_3_ greenschist facies overprint are not sufficiently quantified to constrain the retrograde P‐T path. The low Al and high Si contents of actinolite do not meet calibration requirements for amphibole‐plagioclase thermometry or Al‐in‐amphibole barometry (e.g., Al ≥ 0.5 apfu and Si ≤ 7.8 apfu). However, retrograde amphibole Al_2_O_3_ contents are consistently below 5 wt% which is consistent with ∼4–7 kbar and 350–450°C from synthesis experiments (Figure 10; Apted and Liou, 1983), and with thermodynamic calculations constraining the amphibolite‐to‐greenschist transition for a typical MORB composition (Diener et al., 2007; Diener & Powell, 2012). TiO_2_ values in retrograde actinolite are also similar regardless of sample depth. These observations suggest that, except for the lower ∼10 m of the BT‐1B core (represented by sample 295), the middle ∼60 m thick section of meta‐mafic rocks exhumed along similar P‐T paths (Figure 12d).

Titanite growth during decompression is common in subducted rocks, as evidenced in many HP/LT terranes by lineation‐parallel overgrowths on rutile, and can efficiently redistribute high‐field strength elements (Lucassen et al., 2010). We interpret the titanite U‐Pb age from sample 209 to record syn‐D_3_ titanite growth during exhumation at ∼90 ± 6 Ma. Again, we emphasize that calculated U‐Pb ages are volumetrically averaged titanite crystallization ages; the relatively large uncertainty for the age in part reflects the low U content but might also reflect the protracted nature of D_3_. Since titanite is syn‐kinematic and could be reactive along the entire inferred P‐T trajectory for the LT sole, the higher age uncertainty might indicate the presence of older intergrown syn‐D_2_ titanite during protracted (re‐) crystallization of syn‐D_3_ titanite. The presence of inclusion‐rich titanite cores and inclusion‐free rims in sample 209 suggests protracted or multistage titanite growth, but analytical uncertainties do not permit us to resolve the timing of these stages with the present data set, nor can we confidently determine whether ages are skewed toward core or rim growth.

Metamorphism became increasingly static and deformation increasingly brittle during D_3_ decompression and cooling. Ductile fabrics were crosscut first by D_3*b*_ reverse‐sense brittle faults under hydrous conditions as evidenced by growth of chlorite, and later by D_3*b*_ normal‐sense brittle faults associated with the influx of CO_2_‐rich fluids that led to the precipitation of calcite in veins (cf. Figures 4c and 4e). Younger normal‐sense micro‐faults decorated with calcite could indicate that some exhumation occurred by brittle extension after underthrusting of the Arabian continental margin. Some CO_2_‐rich fluids could have been sourced locally from calcareous strata in the metasedimentary section or from the underlying carbonate platform sediments (cf. de Obeso et al., 2018).

### 6.3 On the Relative Timing of Listvenite Formation

The source of CO_2_‐rich fluids responsible for extensive listvenitization in the overlying mantle peridotite section and the timing and mechanisms of mass transfer, are matters of major scientific interest. While some workers have proposed that CO_2_ was derived from the metamorphic sole as is present in BT‐1B (Lafay et al., 2019), this inference is not supported by our structural and petrologic observations. Several billion metric tonnes of CO_2_ are required for near‐complete carbonation of several km thick peridotite mantle (Kelemen et al., 2011; Kelemen & Manning, 2015). However, the volume of carbonate in the sub‐ophiolite metamorphic rocks present in BT‐1B is minimal (<1%) and typically localized to discontinuous veinlets and thin carbonate‐rich layering metamorphosed into calc‐phyllite. An abundance of titanite in calcite‐bearing metasedimentary rocks implies extremely high X_H2O_ fluids (Kohn, 2017) during both subduction and exhumation since even a small component of CO_2_ in a fluid would have stabilized rutile with calcite. Furthermore, Sr isotopic values of BT‐1B sub‐ophiolite metamorphic rocks are very different from Sr signatures of the overlying listvenitized mantle, but the listvenite mantle is isotopically similar to the underthrust unmetamorphosed Hawasina sedimentary strata (de Obeso et al., 2018). Together these observations indicate that the calcite‐filled brittle micro‐faults in the BT‐1B metamorphics formed prior to tectonic juxtaposition of the metamorphic sole with the ophiolite along the Basal Fault. Thus, listvenitization occurred sometime before juxtaposition with the metamorphic sole sampled in the BT‐1B core. We conclude that the metamorphic sole rocks in the BT‐1B core were not the source of CO_2_ for mantle carbonation.

The Basal Fault exposed in the BT‐1B core is a ∼40‐cm‐thick brittle cataclastic zone. The sense and amount of offset across the zone is unknown, but the metamorphic rocks in the core record small‐scale normal offsets suggestive of roughly horizontally directed extensional deformation. As these rocks were not the source of the CO_2_ that listvenitized the overlying mantle rocks, significant km‐scale displacement is plausible to explain the mantle‐sole juxtaposition present in the BT‐1B core.

### 6.4 Summary

The structural petrology and geochronology data presented above indicate that D_1_ and D_2_ developed during prograde‐to‐peak subduction, and penetrative ductile deformation occurred under epidote‐amphibolite facies conditions (∼7–10 kbar, 490–540°C) at ∼91 Ma. D_3_ developed as mechanical behavior changed from pervasively ductile strain to increasingly localized brittle behavior as retrograde temperatures lowered to greenschist facies conditions at ∼90 Ma. D_3_ is concurrent with the initial stages of exhumation, which we interpret to have occurred along the top of the subduction channel shear zone. During the deformation progression from D_1−3_, older veins were transposed into foliation parallel layering, and cross‐cut by later veins, indicating that the high fluid pressure conditions required for extension fracturing was present, at least transiently, during prograde and retrograde metamorphism. Greenschist overprinting did not completely wipe out evidence of syn‐subduction prograde‐to‐peak conditions, and carbonate minerals are volumetrically minor, indicating that the water flux and CO_2_ content were limited, respectively.

## 7 Discussion and Implications

### 7.1 Tectonic Significance of the LT Sole

**Figure 13:**
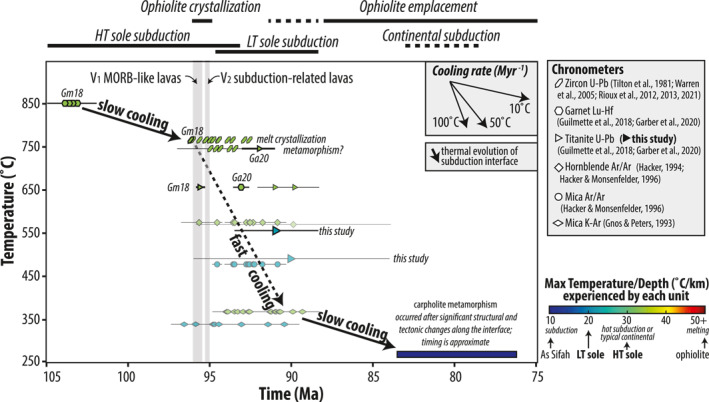
Temperature‐time and tectonic evolution of the Samail ophiolite and metamorphic sole. Ages are compiled from references shown on the right‐hand panel. Symbol colors correspond to different geothermal gradients that each unit experienced during formation, for example, progressive subduction of the HT sole (green), followed by LT sole (teal), and finally the Arabian continental margin (dark blue). Progression from warmer to cooler colors in subduction‐related rocks illustrates interface cooling through time. Data sets have different geological significance (e.g., crystallization vs. cooling) and uncertainties (discussed in the text), but in general suggest a slow‐fast‐slow cooling pattern during subduction infancy. Ar/Ar and K‐Ar data sets are faded in the background to emphasize higher precision chronometers with clearer tectonic contexts shown in bold colors. Gu18 = Guilmette et al. (2018) and Ga20 = Garber et al. (2020). New titanite results from this study are labeled.

The LT sole is commonly envisioned as a section of the down‐going slab that subducted at the same time as the HT sole, but was accreted at shallower depths. Our petrological analysis paints a contrasting picture and leads us to conclude that this LT sole section reached similar pressures (i.e., depths) as the HT sole (∼30 km), but experienced ∼300°C colder peak temperatures. Ductile strain during dynamic epidote‐amphibolite facies metamorphism pervasively sheared the mafic and sedimentary layers forming the LT sole. Our new titanite U‐Pb data indicate that this occurred at ∼91 ± 2.5 Ma, which is distinctly younger than zircon crystallization in HT sole leucocratic lenses, garnet growth in the HT sole, and plagiogranite crystallization in the ophiolite (Figures 12 and 13). Together these data provide new constraints on rates of interface refrigeration (quantified below) over the first ∼10–15 Myr of this subduction zone's lifetime, which partly occurred in proximity to a proto‐arc spreading center.

Initial exhumation of these rocks from maximum depths of 30 km implies ∼15 km of return flow along the interface assuming a ∼15–20 km thickness of the ophiolite before erosional denudation; otherwise, extensional thinning of the ophiolite above these metamorphic rocks was >50%. If the contact beneath the ophiolite dips at ∼25°, then the minimum up‐dip return flow along the plate interface would be ∼40 km, and less if steeper (Figure 12d). Within the LT sole, metamorphism became increasingly static and deformation increasingly brittle, as the rocks approached depths of ∼15–20 km during return flow (Figures 10 and 12). Our observations suggest that juxtaposition of this section of LT sole with the base of the HT sole was probably concurrent with exhumation of these rocks from depths of ∼15 km and was accommodated by dominantly brittle structures.

The present contact between LT sole rocks and the ophiolite in the BT‐1B core is a localized brittle fault zone. This fault contact may have originally been a ductile thrust that juxtaposed different units in the subduction channel during syn‐subduction exhumation, but we interpret its most recent operation as a detachment fault at the base of the ophiolite that accommodated a later, brittle stage of exhumation of the metamorphic sole. Detachment faulting locally cut out the HT sole section.

### 7.2 Stepwise Underplating or Continuous Changes in P‐T?

In the meta‐mafic rocks, maximum Al_2_O_3_ and TiO_2_ values in amphibole (interpreted as indicators of peak P and T, respectively) and semi‐quantitative constraints from multi‐equilibrium thermobarometry show a slight decrease with depth down the BT‐1B core (Figures 8 and 11). Even with our dense data set, it is difficult to discern whether changes in inferred maximum T are continuous as expected for conductive cooling across a compositionally heterogeneous shear zone (Garber et al., 2020; Hacker & Mosenfelder, 1996) or rather occur as stepwise changes as expected for tectonic juxtaposition of several discrete P‐T‐X slices (cf. Soret et al., 2017).

The latter interpretation of discrete tectonic slicing is qualitatively supported by the observation of three petrologically distinct subsections in the core described above, comprising upper metasedimentary phyllites (Section 1), a middle epidote‐rich meta‐mafic section (Section 2), and a lower amphibole‐rich meta‐mafic section (Section 3). Furthermore, our new titanite data suggests the LT sole was subducted after the HT sole, implying that each structural unit may record distinct snapshots of the infant interface throughout its early evolution during progressive subduction and punctuated underplating.

However, within the LT sole rocks studied here, upper Section 1 appears to have reached slightly higher P but similar peak *T* (Figures 8a and 8b, 11) to middle Section 2 , which could instead reflect a continuous increase in P along a dipping, roughly slab‐parallel ∼500°C isotherm (illustrated schematically in Figure 12c). Recently, Garber et al. (2020) presented phase equilibria modeling and Lu‐Hf garnet ages from a metasedimentary rock collected ∼12 m beneath the peridotites at Wadi Tayin, and concluded that it records lower peak P‐T (∼7.5 ± 1.2 kbar and 665 ± 32°C) and may have metamorphosed later (∼93 Ma) than the structurally higher meta‐mafic rocks (∼11–13 kbar and 800–900°C at ∼103–104 Ma (Cowan et al., 2014; Guilmette et al., 2018; Soret et al., 2017). If the older garnet ages are accurate, this could be taken as evidence for semicontinuous changes in peak P‐T along the interface, wherein different rock types experienced different peak conditions, but similar geothermal gradients (∼18–25°C/km; Figure 10) over a protracted period of time (104–93 Ma).

It is possible that differences in observed amphibole chemistry could also reflect bulk composition as opposed to P‐T conditions. The maximum Al‐content in sample 295 (Section 3), for example, is similar to the middle section samples, but (Na + K) and TiO_2_ values are lower on average than the rest of the core. While this could indicate the bottom of the core occupied a structurally deeper position within the shear zone across a continuous P‐T gradient and/or is a tectonic slice that was subducted and accreted under cooler conditions (cf. Ernst & Liu, 1998; Laird & Albee, 1981; Spear, 1995), it could also reflect metamorphism of a compositionally distinct protolith with slightly less Na, K, and Ti (see Table S1 in Supporting Information S1). For comparison, HT sole rocks also record a decrease in Ti‐in‐amphibole with structural depth away from the peridotite mantle, from ∼2.3 to ∼1.0 wt% TiO_2_ (Ambrose et al., 2021; Cowan et al., 2014; Ghent & Stout, 1981; Gnos, 1998; Searle & Malpas, 1982; Soret et al., 2017). Soret et al. (2017) cited this observation as evidence for “tectonic slicing” during subduction, but Garber et al. (2020) pointed out that this could instead reflect elevated bulk TiO_2_ in amphibolites closer to the Samail Thrust (see Ishikawa et al., 2005) rather than discrete jumps in peak P‐T. Since the 200–250 m thick LT (“fine‐grained amphibolite”) section at Wadi Tayin has a consistent bulk composition throughout (Ishikawa et al., 2005), and variations in bulk rock chemistry reported here (*n* = 4) are within the reported range of the Ishikawa et al. (2005) data set, we prefer the interpretation that differences in peak amphibole chemistry are primarily a function of P‐T. More bulk rock geochemical analysis and petrologic modeling is needed to address this further.

It is reasonable to envision that sections of the subducting slab that reached different peak P‐T along a continuous geotherm at a given time were subsequently juxtaposed by post‐peak ductile shearing and/or semi‐brittle displacements in a ”stepwise” fashion. Therefore, we suggest that the HT‐LT sole sequence may be constructed by stepwise tectonic slicing and underplating on ∼million year timescales, but at any given point in time an interface slice may record continuous P‐T gradients that are characteristic of the subduction zone's thermal structure at that time (∼20–30°C for the HT sole (Garber et al., 2020) and ∼15–22°C for the LT sole (this study; Figure 11). Within a given interface slice, structural juxtaposition of rocks that were originally separated by several tens of kilometers depth at respective peak conditions can be accomplished by large magnitudes of ductile shearing during exhumation (see Figures 12c and 12d; cf. Garber et al., 2020).

### 7.3 Evidence for a Multistage Cooling History During Subduction Infancy

In the broader framework of subduction infancy, the earliest episode of deformation and metamorphism is recorded in the HT sole. The early history of the HT sole remains contentious, and our study does not provide new constraints on its evolution. HT sole protoliths were dominantly basaltic and probably reflect subduction of distal seafloor with limited sediment cover, far from the Arabian continental margin (cf. Soret et al., 2017) (Figure 12a). Alternatively, incoming sediment could have been scraped off at shallower depths during initial convergence or melted out at high temperature conditions (Dubacq et al., 2019; Garber et al., 2020). The HT sole subducted to peak P‐T conditions of ∼10–14 kbar (∼35–45 km) and ∼750–850°C, consistent with a temperature‐depth trajectory of ∼20–30°C/km (Cowan et al., 2014; Searle et al., 2015; Soret et al., 2017). According to Lu‐Hf dating of three samples from two locations reported by Guilmette et al. (2018), garnet growth and peak temperatures in the HT sole might have occurred as early as ∼103–104 Ma (Figures 10, 12 and 13), which predates igneous crystallization of the ophiolite plagiogranites at ∼96–95 Ma by ∼6–8 Myr. Furthermore, Garber et al. (2020) presented Lu‐Hf garnet ages and equilibrium P‐T modeling from the structurally highest metasedimentary rock documented at Wadi Tayin (12 m beneath peridotites), and concluded that latest stages of garnet growth occurred ∼93 Ma at 7.5 ± 1.2 kbar and 665 ± 32°C.

Taking the ∼103 Ma garnet ages from Guilmette et al. (2018) as records of peak metamorphism of the uppermost meta‐mafic HT sole, then initial cooling near the new plate interface from ∼850°C to ∼750°C appears to have occurred very slowly, proceeding at rates of ≤10–20°C/Myr (Figure 13). This suggests that initial underthrusting, which predated formation of the crustal section of the ophiolite, was very slow. Comparable geothermal gradients in HT meta‐mafic rocks and HT metasedimentary rocks demonstrate that the infant interface stayed relatively hot for ∼2–3 Myr after ophiolite formation at ∼96–95 Ma, through ∼93 Ma.

Following the initial stage of slow convergence and slow cooling, existing metamorphic geo‐ and thermochronology suggest the interface experienced a stage of rapid cooling between ∼93–90 Ma (lagging behind ophiolite formation by ∼2–3 Myr and lasting for ∼3 Myr). This distinct stage of faster cooling may indicate faster subduction, on the order of several cm/yr (cf. Holt & Condit, 2021; Peacock & Hyndman, 1999; van Keken et al., 2002). Rapid cooling from near‐peak T through ∼750°, 650° and 550°C is captured by zircon U‐Pb crystallization ages in the HT sole leucosomes (Rioux et al., 2016; Roberts et al., 2016; Styles et al., 2006; Warren et al., 2005), titanite U‐Pb cooling ages (Guilmette et al., 2018), titanite U‐Pb crystallization ages (this study and Garber et al., 2020), and hornblende ^40^Ar/^39^Ar cooling ages (Hacker, 1994), respectively (Figure 13). Hornblende ^40^Ar/^39^Ar plateau ages of ∼94 Ma and K‐Ar ages for muscovite of ∼92 Ma are only slightly younger than the plagiogranites (Hacker et al., 1996). Assuming Ar closure temperatures of ∼550°C and ∼400°C for hornblende and white mica, respectively, the HT sole records cooling rates of ∼75°C/Myr between 95 and 92 Ma (Figure 13). However, some of these older cooling ages that predate our new titanite U‐Pb crystallization ages may be affected by excess Ar. Taking the youngest white mica Ar‐Ar and K‐Ar ages, cooling rates between ∼93 and 90 Ma could have exceeded 100°C/Myr (Figure 13).

Multistage cooling of the infant subduction interface is supported by our new petrologic and geochronologic data that demonstrate the LT sole sequence reached peak P‐T conditions of ∼7–10 kbar (∼25–35 km) and 500°C at ∼91 Ma (Figures 12c and 13). Peak P‐T conditions are consistent with a depth‐temperature trajectory of ∼15°C/km, which is distinctly colder than ∼25°C/km at 103 Ma (Figure 10) and suggests the thermal structure of the subduction zone changed drastically. Cooling appears to have occurred concurrently with changing physical properties of the interface, since the protoliths for the LT sole were different from those for the HT sole. The LT sole comprises dominantly sedimentary lithologies and basalts, which could reflect increasing sediment input with proximity to the continental margin (Figure 12a).

During the rapid refrigeration stage, overlapping cooling ages indicate that both the HT and LT sole units were mechanically decoupled from the descending plate, by underplating or accretion, while fast subduction proceeded and the forearc block continued to refrigerate. It is important to emphasize that, because this young subduction zone was actively refrigerating, the fact that the HT and LT metamorphic soles experienced simultaneous cooling does not necessarily imply that these units were exhuming (i.e., decompressing) and flowing up the subduction channel shear zone. This inference is only possible by integrating geo‐/thermochronology with structural petrology, which reveals fabric regeneration in LT sole rocks under lower P and T conditions at ∼90 Ma (e.g., reduction of Si‐in‐phengite values and actinolite overgrowths on hornblende; Figure 6). Exhumation‐related deformation appears to have been preferentially localized toward the structural base of the metamorphic sole package, imparting a pervasive greenschist facies overprint to wetter, weaker, finer‐grained LT sole rocks, while structurally higher HT sole rocks largely escaped deformation and metamorphism under greenschist facies conditions during return flow (Figure 12d; cf. Soret et al., 2017; Soret et al., 2019).

Rapid cooling was subsequently followed by a renewed stage of slower cooling on the order of ∼10–20°C/Myr for another 10–15 Myr (Figure 13). Cooling proceeded as the subduction zone consumed old, cold oceanic lithosphere and was associated with large‐scale tectonic and structural changes before continental margin subduction. In other ophiolite localities, sole metamorphism has been shown to culminate in blueschist facies conditions. For example, amphibolite facies meta‐basalts in the Kiziltepe ophiolite in Turkey and the Newfoundland Appalachians record core‐to‐rim zonations from hornblende to glaucophane or crossite (Dilek & Whitney, 1997; Jamieson, 1977; Plunder et al., 2016). In Oman, blueschists and eclogites crop out SE of Muscat (Figure 1) and reveal that continental margin subduction eventually reached P‐T conditions of ∼20 kbar and ∼500°C (Searle et al., 1994; Wendt et al., 1993) at ∼80 Ma (Warren et al., 2003). Therefore, interface cooling eventually achieved a thermal structure that is typical of established subduction zones—∼7°C/km—before the Arabian platform jammed the trench (Figures 10 and 13).

### 7.4 Significance of Cooling for Interface Mechanics and Early Subduction Dynamics

The formation of the epidote‐amphibolite facies LT sole at ∼91 Ma under a depressed geothermal gradient (relative to the HT sole; illustrated by cooler data colors from HT to LT sole in Figure 13) provides direct insight into evolving interface mechanical properties during subduction infancy. Interface refrigeration is expected where subduction of old, cold lithosphere is continuous and fast (Agard et al., 2020; Cloos, 1985; Hacker et al., 1996; Platt, 1975). This is especially true if subduction continues after ophiolite formation, since the spreading center—which serves as a heat source for high‐temperature metamorphism—migrates laterally away from the developing interface at the half spreading rate (∼10 cm/yr spreading rate for ≤1 Myr in Oman). Cooling leads to changes in metamorphic mineral parageneses and increased depths of prograde metamorphic dehydration of the shear zone (Holt & Condit, 2021; Poli & Schmidt, 1995; van Keken et al., 2011). Both of these phenomena strongly impact the operative microphysical deformation mechanisms along the interface. In general, the temporal progression in metamorphic rock stability from garnet‐granulite, to garnet‐amphibolite, to epidote‐amphibolite at a given depth should lead to profound weakening. Compared to dry, coarse‐grained garnet‐ and pyroxene‐bearing rocks, wet, finer‐grained plagioclase‐rich amphibolites are weaker (Ambrose et al., 2021; Getsinger et al., 2013; Getsinger & Hirth, 2014; Soret et al., 2019) and may effectively localize strain within the new plate boundary shear zone during subduction infancy.

Rheological weakening along the interface can control upper plate stress state and may reduce mechanical coupling to a point where upper plate extension can occur (e.g., Androvičová et al., 2013; Čížková & Bina, 2013; Lallemand et al., 2005). In Oman, HT sole cooling (and rehydration?) and ophiolite formation appear to have occurred concurrently or in immediate succession (Figures 12c and 13). These observations may point to a fundamental feedback between interface cooling, rheological weakening via hydration and/or grain size reduction, mechanical decoupling, and upper plate extension, leading to forearc/proto‐arc spreading. Continued underthrusting after ophiolite formation rapidly refrigerates the forearc and leads to epidote‐amphibolite facies metamorphism that generates colder, wetter, finer‐grained, and therefore weaker rocks that facilitate continued strain localization. We suggest that the HT sole and LT sole slices record the transition from forced to self‐sustaining subduction, respectively, and therefore capture an increase in convergence rate, rapid decrease in interface temperature, and increase in cumulative plastic strain along a lithosphere‐scale plate boundary shear zone (see Hall et al. (2003) and Gurnis et al. (2004) for finite element simulations of this transition). As such, it is crucial to better quantify the rheological properties of polyphase epidote‐amphibolite facies rocks of the LT sole, in order to understand the evolution in strength and deformation mechanisms that facilitate this pivotal geodynamic stage in the development of a subduction zone.

Finally, the expected evolution in metamorphic and mechanical properties along the interface with interface cooling is consistent with documented trends in ophiolite lava chemistry. In ophiolitic crust, there is a well‐documented progression from relatively dry, MORB‐like volcanism to hydrous boninitic volcanism, to locally calc‐alkaline, with stratigraphic height and time (Leng et al., 2012; Stern et al., 2012; Whattam & Stern, 2011). For Oman, Rioux et al. (2012) presented high‐precision U‐Pb zircon geochronology to bracket the transition from “V1” MORB‐like volcanism at 96.1–95.6 Ma to “V2” subduction‐influenced volcanism at 95.2–95.0 Ma. These geochemical changes mirror evolving metamorphic characteristics of the interface, from hot and dry during HT sole subduction, to partially melted at peak HT sole conditions, to cooler and wetter during LT sole subduction. The high‐precision temporal constraints on V1‐V2 transition in ophiolite chemistry presented by Rioux et al. (2021) may therefore also reflect the forced to self‐sustaining transition in the infant subduction zone beneath the ophiolite.

## 8 Conclusions

Low‐temperature metamorphic soles (LT soles) are high‐strain subduction shear zones in tectonic contact with the base of large‐slab ophiolites that contain vital information about rates of cooling, metamorphic conditions, and progressive strain localization during subduction infancy. Compared to their structurally higher, thinner, garnet‐bearing, HT metamorphic soles counterparts, LT soles have been severely understudied. One hundred and four continuous meters of LT sole were recovered during OmanDP drilling at Site BT‐1B, in the footwall of the brittle Samail Thrust (i.e., Basal Fault). Following OmanDP core logging, we scrutinized the 104 m section with high sampling density (36 samples collected and 12 studied in detail) compared to the average field‐based study using a combination of structural, microstructural, petrologic, and geochronologic techniques. The dense sample suite revealed a complex history of subduction‐ and exhumation‐related deformation and metamorphism and allowed us to quantify interface cooling rates over the first ∼10–15 Myr of this subduction zone's lifetime.

In this section of LT sole, upper phyllitic metasedimentary (34 m) and lower schistose meta‐mafic rocks (70 m) record two stages of ductile deformation and metamorphism during subduction to epidote‐amphibolite facies conditions (D_1−2_). All lithologies were then variably overprinted by ductile greenschist facies retrogression (D_3_) in a subduction channel configuration. Our observations suggest that tectonic juxtaposition of this section of LT sole with the HT sole involved ductile return flow that accommodated vertical translations of perhaps 10–20 km, thus bringing rocks from maximum depths of ∼25–35 km to the base of the overriding ophiolite mantle. Distributed, exhumation‐related ductile strain evolved to localized brittle chloritized microthrust faulting, followed by calcite‐filled normal‐sense microfaulting, consistent with kinematic changes associated with exhumation and extensional denudation of the ophiolite.

Amphibole mineral zonations and Si‐in‐phengite values evolve systematically with progressive fabric development and constrain changes in P and T during subduction and return flow. The relative timing of deformation inferred from crosscutting fabrics is consistent with absolute timing constrained by U‐Pb ages of syn‐kinematic titanite growth. Prograde‐to‐peak fabrics record mineral chemistry that indicates the LT sole reached peak P‐T conditions of ∼7–10 kbar and ∼450–550°C. Titanite U‐Pb ages constrain the timing of peak fabric development to ∼91 Ma. Dynamic greenschist overprinting occurred as P and T decreased and became progressively less pervasive by ∼90 Ma. The timing of peak LT sole deformation and metamorphism postdates recent garnet Lu‐Hf crystallization ages in the uppermost meta‐mafic HT sole by ∼12–13 Myr, garnet Lu‐Hf crystallization ages in the structurally highest metasedimentary lens at Wadi Tayin by ∼2–3 Myr, zircon U‐Pb ages from crystallized partial melt lenses in the HT sole by ∼3–4 Myr, and plagiogranite crystallization in the ophiolite crust by ∼4–5 Myr.

Our new petrologic and geochronologic data from detailed study of the 104 m LT metamorphic sole section sampled in the BT‐1B drill core show that these rocks reached similar peak pressures (i.e., depths) as the structurally higher, garnet‐bearing, HT metamorphic sole found elsewhere in Oman, but experienced ∼300°C colder peak temperatures, and subducted later. These data support a revised tectonic model of progressive subduction, punctuated underplating, and return flow of the metamorphic sole beneath the Samail Ophiolite while the infant interface was refrigerating. All of these dynamic processes occurred during subduction infancy, prior to ophiolite emplacement on the Arabian continental margin. Subduction infancy was characterized by a multistage, “slow‐fast‐slow” cooling history. We suggest that the first slow‐fast switch marks the fundamental transition from forced to self‐sustaining subduction, as the subduction zone evolved toward its characteristic steady‐state thermal structure, which is consistent with inferred mechanical changes in a colder, wetter, and finer‐grained subduction shear zone. This study affirms that low‐temperature metamorphic sole rocks record crucial details regarding the first few million years of a subduction zone's lifetime, warranting fresh investigation of fine‐grained LT sole rocks beneath other large‐slab ophiolites around the world.

## Supporting information

Supporting Information S1

Data Set S1

Data Set S2

## Data Availability

Core sample IGSNs (IEBTB000O, IEBTB000M) are in the SESAR database (https://app.geosamples.org/sample/igsn/IEBTB000M&header=1; https://app.geosamples.org/sample/igsn/IEBTB000O&header=1). Bulk geochemistry and electron microprobe data in this manuscript are available on the EarthChem repository (https://doi.org/10.26022/IEDA/111813) and titanite U‐Pb geochronology is available on Geochron (https://www.geochron.org/) and in the Supplementary Material.
