# Supplementary material for: Structural and Thermal Evolution of an Infant Subduction Shear Zone: Insights From Sub‐Ophiolite Metamorphic Rocks Recovered From Oman Drilling Project Site BT‐1B
Source: J Geophys Res Solid Earth. 2021 Dec 4;126(12):e2021JB021702. doi: 10.1029/2021JB021702 (PMC9285456; doi:10.1029/2021JB021702)
Supplement: Supplementary file 1 — Supporting Information S1 [file JGRB-126-0-s002.pdf]

JGR Solid Earth

Supporting Information for

Structural and thermal evolution of an infant subduction shear zone: Insights from sub-ophiolite metamorphic rocks recovered from Oman Drilling Project Site BT-1B

Alissa J. Kotowski [1,2•], Mark Cloos [1], Daniel F. Stockli [1], Eytan Bos Orent [1,3]

[1] Department of Geological Sciences, Jackson School of Geosciences, University of Texas at Austin, USA

[2] Department of Earth and Planetary Sciences, McGill University, Montreal, Canada

[3] Department of Geosciences, University of Arizona, USA

* Now at Department of Earth Sciences, Utrecht Unversity, Utrecht, The Netherlands

Contents of this file

Text S1 to S4
Figures S1 to S7
Tables S1 to S5

Additional Supporting Information (Files uploaded separately)

Captions for Datasets S1 and S2

Introduction

This files contains supporting text, figures, tables, and captions for datasets for bulk geochemical and mineralogical analyses (XRF, XRD), shipboard core logging methods used by the OmanDP Science Party while onbard Chikyu, post-shipboard optical petrographic and quantitative electron microprobe analysis (EMPA), results from multi-equilibrium P-T calculations, and U-Pb titanite LA-ICP-MS data and strategies for data processing.

Text S1. Bulk Geochemistry and Metamorphic Mineralogy

Bulk rock geochemistry: Bulk geochemistry for one meta-sediment, one intercalated meta-sedimentary and meta-mafic, and three meta-mafic samples was obtained. Samples were processed by Franklin & Marshall College lab manager Dr. Stanley Mertzmann. Homogeneous ~5-8 g samples (as best as possible considering limitations of sample size) were selected from shipboard quarter cores for analysis. Details of the laboratory procedure are summarized briefly herein.

Analyses were done using the Panalytical PW 2404 X-Ray Flourescence spectrometer at Franklin & Marshall College. Total volatiles were fist determined by placing the sample in a muffle furnace at 950˚C for 1.5 hours, desiccating the sample, and comparing pre-

and post-dessication weights. Anhydrous sample powders were then mixed with lithium tetraborate, transferred to a platinum crucible, heated until molten, and quenched to produce a glass disk for major element (SiO_2, TiO_2, Al_2O_3, Fe_2O_3 (total), MnO, MgO, CaO, Na_2O, K_2O, P_2O_5) XRF analysis. Ferrous Fe was determined using a modified Reichen et al. (1962) method. Trace element analysis was accomplished by mixing powders with a copolywax powder and pressing the mixture into a briquette (reported in ppm for Rb, Sr, Y, Zr, V, Ni, Cr, Nb, Ga, Cu, Zn, Co, Ba, U, Th, La, Ce, Sc, and Pb). Working curves for each element are determined by analyzing geochemical standards prepared as above; data have been synthesized by Abbey (1983) and Govindaraju (1994).

X-Ray Diffraction: XRD was performed on the Bruker D8 Advance X-Ray Diffractometer in the Department of Geological Sciences at the University of Texas at Austin. Following visual characterization (and complemented by optical petrography of corresponding thin sections, described below) of shipboard quarter core samples, all visually distinct metamorphic layers and veins were micro-drilled into a powder using a fixed diamond-tipped drill. The powder was transferred to a mortar and pestle and ground to uniform fineness. Samples were placed on circular glass XRD mounts. Powdered samples were suspended in one drop of acetone and spread along the surface to produce a coat of even-thickness along the glass slide.

XRD patterns were collected for 142 micro-drilled layers and veins (68 meta-mafic layers, 22 meta-sedimentary layers, 49 veins of variable orientation with respect to the foliation, and 3 listenvites). Samples were analyzed through a 2-theta values of 4-65°. Patterns were stripped of background counts, refined with Fourier smoothing, and adjusted for x-offset where necessary using the Bruker EVA software. Identification of phases was accomplished using the ICDD PDF-2 Minerals database. Peaks were labeled for minerals that were properly identified and, where possible, confirmed petrographically. XRD sample preparation and analysis was performed by undergraduate student Eytan Bos Orent, under supervision of Kotowski and Cloos.

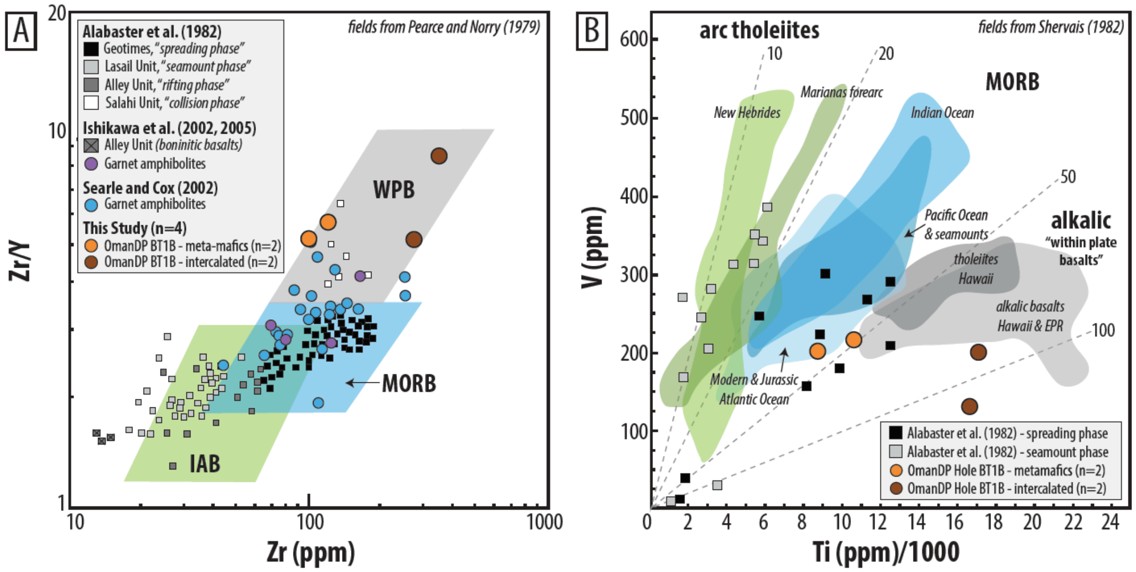

Figure S1. Bulk rock geochemistry from BT-1B core samples in orange and brown circles, compared to published values for ophiolite crust and the HT metamorphic sole. (A) Zr vs. Zr/Y discrimination diagram, following Pearce and Norry (1979). WPB = within-plate basalts, MORB = mid-ocean ridge basalts, IAB = island-arc basalts. (B) Ti vs. V discrimination diagram after Shervais (1982). Colored polygons encompass published geochemical data for lavas from various tectonic settings and geographic locations as indicated.

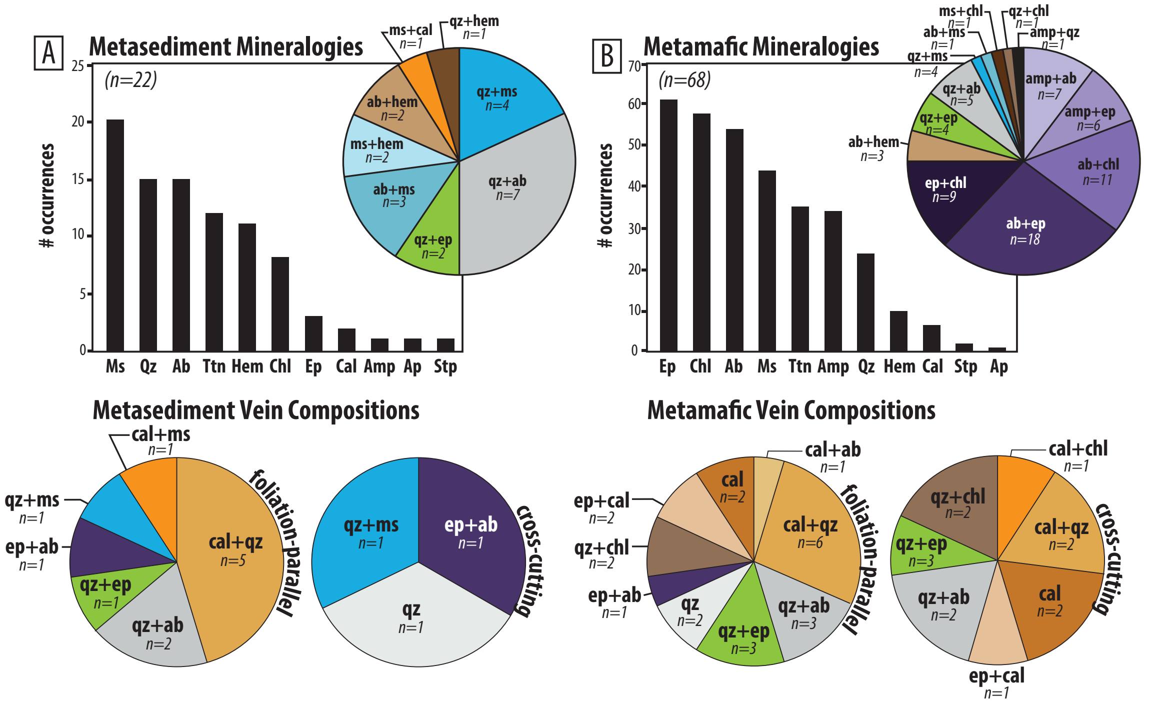

Figure S2. Results from XRD analyses of micro-drilled compositional layers for metasediments (A), and meta-mafics (B). All visually distinct layers were sampled in order to characterize petrologic variability. Results for each individual micro-drilled sample are shown in Supplementary Table A3. In (A-B), “n" indicates the number of samples analyzed. Pie charts show the number of occurrences of specified mineral pairs, representing the two most abundant phases in the sample, estimated from the relative intensities of X-Ray peaks.

Text S2. Shipboard Core Logging Methods

BT-1B was visually and instrumentally described according to Integrated Ocean Drilling Project (IODP) protocols on board the Japanese drillship Chikyu in August-September 2017. This particular contribution focuses on post-Chikyu structural and petrologic characterization in the lowermost 104 m of the BT-1B core.

Onboard Chikyu, we characterized host rock lithology, taking note of colors and mineralogy, as well as distributions and types of veins cutting the host rock. Vein descriptions included mineralogy and modal proportions, grain size, thickness, and morphology. Host rock structure, which is summarized briefly in Section 3, was described as massive, brecciated, cataclastic, “fragmented by veins" (i.e. contains brecciated veins), or foliated; localized structures were described as faults (mm-to-10's of cm scale), cataclasites (mm-to-cm scale), single veins and/or vein sets (and mineralogy), shear zones (mm-to-cm scale), folds, and cracks (i.e. did not contain mineralization or apparent displacement; only documented in listvenites). We documented cross-cutting relationships and relative ages of structural features, stable host-rock and vein mineralogy relative to deformation events, and the downhole abundance and petrologic characteristics of brittle deformation features. Shipboard thin sections were made for first-pass petrologic and micro-structural descriptions.

After shipboard logging, we collected 36 samples from the 104 m of sub-ophiolite metamorphics for analysis at the University of Texas at Austin (8 phyllitic, mica-rich metasediments and 28 epidote- and amphibole-rich meta-volcanics). Samples were collected at roughly even intervals down-core and were selected to represent the core's lithological and structural heterogeneity. Features of special interest were also sampled. Each sample was cut into a microprobe polished 30 um thin section, oriented parallel to lineation and perpendicular to foliation.

Sample ID	AK199	AK209	AK222	AK264	AK295
Lithology	*metasediment*	*intercalated*	*intercalated*	*metamafic*	*metamafic*
**SiO2**	59.91	48.27	50.42	47.31	46.58
**TiO2**	2.13	2.87	2.77	1.76	1.45
**Al2O3**	14.26	17.51	16.53	17.71	16.64
**Fe2O3T**	10.41	14.52	13.14	12.51	11.99
**MnO**	0.10	0.17	0.21	0.17	0.18
**MgO**	1.72	2.22	4.32	3.64	7.48
**CaO**	3.18	4.29	5.58	12.80	12.07
**Na2O**	5.03	1.83	3.33	3.35	2.40
**K2O**	1.91	6.38	2.80	0.63	0.47
**P2O5**	1.12	1.53	0.78	0.29	0.17
**Total**	**99.77**	**99.59**	**99.88**	**100.17**	**99.43**
**LOI**	2.46	3.14	3.50	2.20	3.44
**FeO**	2.93	2.45	7.22	4.37	6.69
**Fe2O3**	7.15	11.80	5.12	7.65	4.56
**Rb**	39.7	109.1	51.8	10.3	8.0
**Sr**	169	157	306	346	468
**Y**	40.4	63.0	41.3	21.1	19.2
**Zr**	270	315	351	120	99
**V**	121	202	129	214	199
**Ni**	29	48	19	64	130
**Cr**	22	11	8	194	307
**Nb**	42.8	52.0	53.1	16.7	13.1
**Ga**	15.0	25.3	23.8	19.0	18.0
**Cu**	12	13	19	132	59
**Zn**	95	168	128	98	86
**Co**	19	30	28	48	49
**Ba**	224	682	386	86	103
**La**	46	68	51	13	6
**Ce**	88	124	113	29	23
**U**	<1	<1	<1	<1	<1
**Th**	4.5	4.4	5.6	<1	1.1
**Sc**	14	24	21	32	33
**Pb**	1	1	4	3	2

Table S1. Major and trace element geochemistry for 1 meta-sedimentary (AK199), 2 intercalated (AK209, AK222), and 2 meta-mafic samples (AK264, AK295). `Intercalated' samples contain both mafic and sedimentary material. Trace element concentrations are reported in ppm.

Text S3. Electron Microprobe techniques and Multi-Equilibrium P-T Calculations

Qualitative X-Ray Mapping: Qualitative X-ray compositional maps were acquired on the JEOL JXA-8200 electron microprobe in the Department of Geological Sciences at the University of Texas at Austin. Polished 30 um thin sections were analyzed using a 15 kV accelerating voltage, focused beam, 50-200 nA current, 1-6 um step size, and 1 ms dwell time. X-ray maps for Si, Al, Ca, Mg, Fe, Na, K, Mn, Ti, and P were collected. Post-processing to produce false color compositional maps was done in ImageJ software by merging element channels with assigned colors.

Quantitative Point Analyses: Quantitative analyses were collected for amphiboles and micas on the JEOL JXA-8200 electron microprobe in the Department of Geological Sciences at UT Austin. Polished 30 um thin sections were analyzed using a 15 kV accelerating voltage, a focused (1 um) beam (for all hydrous phases, due to extremely fine mica grain sizes), 10 nA current, and 30 s counting time for all elements. Synthetic compounds and natural homogeneous minerals were used as standards, and secondary standards were analyzed throughout analytical procedures. Data were processed using the JEOL ZAF procedure. Major element oxide weight percents were converted to cations per formula unit first by calculating water by difference, then assuming 24 and 12 atoms of O for amphibole and white mica, respectively (including H as a cation). Amphibole calculations assigned the T-site a total of 8 cations (Si+Aliv). Remaining Al was assigned to Alvi . Fe2+ , Ca and Na were assigned to the M4 site for a total of 2 cations, and excess Na was assigned to the A site. Ferric and ferrous iron were calculated based on cation normalization to

achieve charge balance. Percent errors of Si elemental analyses were consistently ~0.2%, which propagates to errors of ~0.006 apfu. Therefore, subtle but measurable differences in Si apfu ranging from ~0.05-0.3 apfu appear to record true heterogeneity in mineral composition. Nomenclature and classification was determined following recommendations approved by the Commission on New Minerals Nomenclature and Classification (CNMNC) of the International Mineralogical Association (IMA) (Hawthorne et al., 2012).

	**MINERAL ASSEMBLAGES**								
	**Sample ID**	**lithology**	***quartz***	***albite***	***muscovite***	***epidote***	***chlorite***	***mg-hbl***	***edenite***
**1**	AK-80Z-01-199.25	metasediment	x	x	x	x	r, v, p		
**2**	AK-82Z-01-201.26	metasediment	x	x	x	x, a	r		
**3**	AK-86Z-04-209.35	metasediment	x, v	x	x	x, a	r		
**4**	AK-90Z-01-219.45	intercalated	x	x, r	x	x	x, r, v		x, c
**5**	AK-92Z-01-222.55	intercalated	x	x	x	x, a	x, r, v		x, c
**6**	AK-99Z-04-243.35	metamafic	x, v, p	x, r, p		x, v	x, r, v		x
**7**	AK-103Z-02-253.7	metamafic	v, p	p, r		x, a	r, p, v	o	x, c
**8**	AK-106Z-03-264	metamafic	v	x, p	m	x, a, v	v, p, r		x
**9**	AK-109Z-02-268.85	metamafic	v	x	m	x	x, v, p, r	x	o
**10**	AK-111Z-02-275.35	metamafic	v	x, v, p, ps	ps	x, a, ps	v, p, r	x, o, c	x, c
**11**	AK-112Z-02-278.5	metamafic	v	x, p, ps	ps, m	x, ps	p, r		x
**12**	AK-125Z-01-295.95	metamafic		x, p		x	x, o, ps	x, c	
			pargasite	***actinolite***	***apatite***	***titanite***	***Fe-Ti-O***	***calcite***	***other***
**1**	AK-80Z-01-199.25	metasediment			x		x,b		
**2**	AK-82Z-01-201.26	metasediment			x	x	x,b		
**3**	AK-86Z-04-209.35	metasediment			x	x	x,b		tur, m
**4**	AK-90Z-01-219.45	intercalated	x, c	o	m		b	v	
**5**	AK-92Z-01-222.55	intercalated	x, c				x,b	v	
**6**	AK-99Z-04-243.35	metamafic	o				x, v	v	
**7**	AK-103Z-02-253.7	metamafic	o	o			x,b	v	
**8**	AK-106Z-03-264	metamafic		o			x,b		
**9**	AK-109Z-02-268.85	metamafic		x			x	p, v	
**10**	AK-111Z-02-275.35	metamafic		o				v	
**11**	AK-112Z-02-278.5	metamafic	x				b	v	sulfides
**12**	AK-125Z-01-295.95	metamafic		x, c				v	sulfides

x = present, foliation-forming	c = cross-cuts foliation
v = vein	p = patchy
b = porphryoblasts	a = allanite cores of epidotes
o = rims & overgrowths	m = minor, ≤ 5%
r = replacement	ps = mineral occurs as pseudomorph?

Table S2. Parageneses of samples selected for detailed EMPA and microstructural analyses. Sample IDs are listed in the left column, and are read as follows: AK-core Z-section-depth downhole (m) .

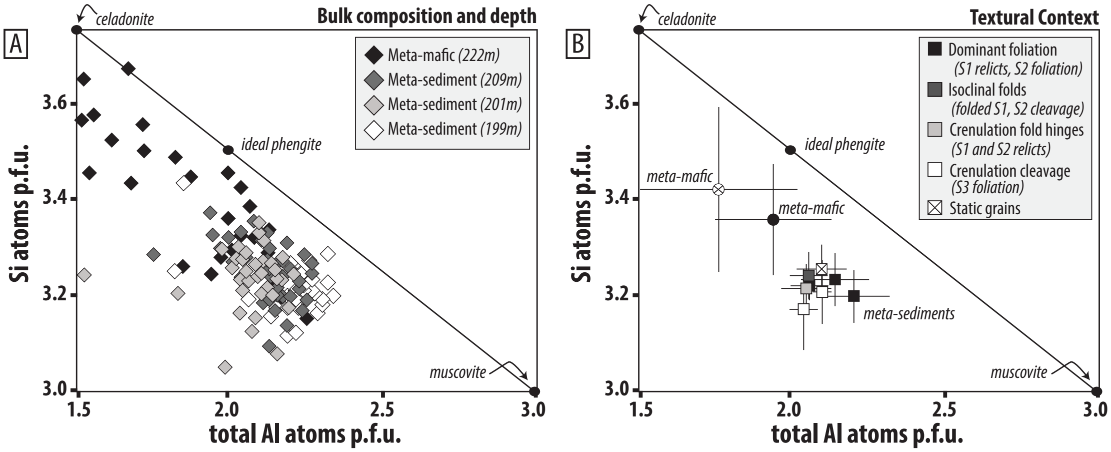

Figure S3. Mica chemistry for meta-sediments (199, 201, 209m) and a meta-mafic sample (222 m). (A) Total Al vs. Si atoms p.f.u. (B) Results grouped by micro-textural context. See text for discussion.

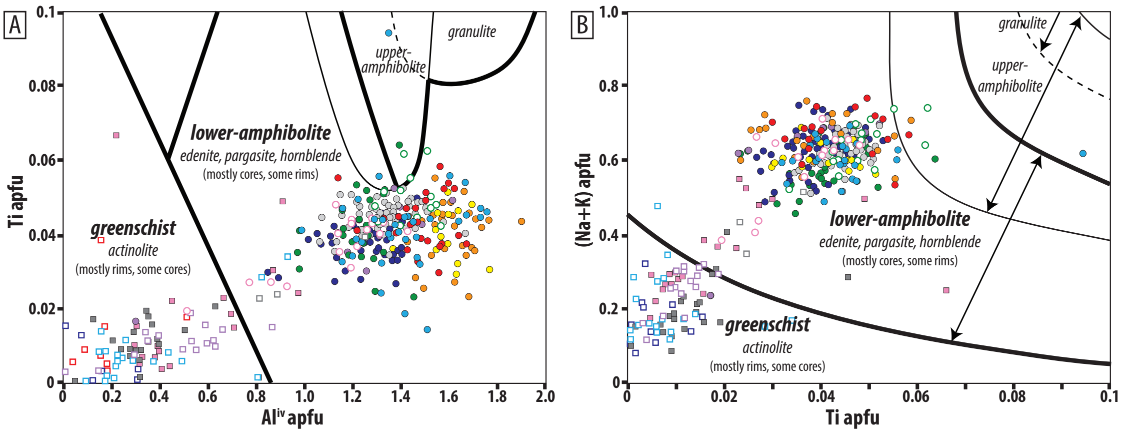

Figure S4. Amphibole point analyses record at least three distinct phases of deformation and metamorphism: prograde D1 greenschist facies, prograde-to-peak D2 lower-amphibolite (epidote-amphibolite) facies, and retrograde D3 greenschist facies. Metamorphic facies fields are from Jin et al. 1991. Color key is the same as Figure 7. (A) Aliv vs. Ti atoms p.f.u., and (B) Ti atoms p.f.u. vs. (Na+K) atoms p.f.u. Greenschist facies amphiboles are actinolite, and lower-amphibolite facies amphiboles are edenite, pargasite, and hornblende.

Multi-equilibrium P-T Calculations: P-T conditions of peak metamorphism were estimated from measured mineral compositions via inverse thermobarometry. Our strategy was based on the amphibole-plagioclase thermometer of Blundy and Holland (1990) and Holland and Blundy (1994), and the Al-in-hornblende barometer of Schmidt (1992). Multi-equilibrium calculations were performed using the Excel spreadsheet from Lawford Anderson, after Anderson (1996). Actinolite cores and/or rims were not considered, because their low Al and high Si contents do not meet calibration requirements (e.g., Al $\geq$ 0.5 apfu and Si $\leq$ 7.8 apfu). Only amphibole compositions that record the maximum P-T conditions recorded by a sample (whether preserved

Texture	*cores*	*rims*	*foliation*	*oblique*	*cores*	*rims*	*cores*	*mantles*	*rims*	*cores*	*rims*	*cores*	*rims*	*cores*	*rims*	*unzoned*	*cores*	*rims*
**Sample**	*AK219*	*AK219*	*AK222*	*AK222*	*AK243*	*AK243*	*AK253*	*AK253*	*AK253*	*AK264*	*AK264*	*AK268*	*AK268*	*AK275*	*AK275*	*AK278*	*AK295*	*AK295*
**#analyses**	*30*	*8*	*37*	*14*	*22*	*16*	*37*	*5*	*21*	*51*	*12*	*21*	*19*	*14*	*16*	*58*	*21*	*3*
**SiO2**	43.19	53.56	43.39	43.32	46.08	44.41	45.05	43.09	53.84	45.56	55.49	51.76	46.44	45.60	52.85	44.45	53.04	48.16
**TiO2**	0.40	0.12	0.38	0.37	0.37	0.44	0.38	0.42	0.08	0.36	0.05	0.18	0.34	0.37	0.11	0.41	0.12	0.26
**Al2O3**	13.96	2.74	14.35	14.09	10.35	11.51	12.11	13.80	2.52	12.19	1.76	5.12	9.70	11.73	4.53	12.63	4.70	10.05
**FeO***	18.03	14.45	16.70	17.06	15.71	17.00	15.64	15.70	13.00	15.40	11.59	13.55	16.90	15.76	11.41	17.57	12.93	14.18
**MgO**	9.51	15.07	9.35	9.08	11.30	10.13	10.96	9.76	14.94	11.12	16.17	14.16	10.62	11.01	15.73	9.89	14.65	11.96
**MnO**	0.30	0.25	0.37	0.43	0.65	0.65	0.20	0.19	0.23	0.23	0.31	0.36	0.36	0.19	0.19	0.19	0.20	0.20
**CaO**	10.57	11.57	10.68	10.69	10.50	10.56	10.88	10.93	11.76	10.95	12.08	11.01	10.66	10.96	11.69	10.87	12.08	11.46
**Na2O**	2.04	0.66	2.09	1.94	1.86	1.96	1.96	2.05	0.72	2.04	0.56	1.15	1.88	1.99	0.83	1.98	0.61	1.35
**K2O**	0.38	0.13	0.28	0.27	0.22	0.44	0.29	0.35	0.09	0.24	0.07	0.16	0.36	0.29	0.15	0.31	0.15	0.28
**F**	0.00	0.00	0.00	0.00	0.00	0.00	0.00	0.00	0.00	0.00	0.00	0.00	0.00	0.00	0.00	0.00	0.00	0.00
**Cl**	0.00	0.00	0.00	0.00	0.00	0.00	0.00	0.00	0.00	0.00	0.00	0.00	0.00	0.00	0.00	0.00	0.00	0.00
**Sum**	98.37	98.55	97.59	97.26	97.04	97.10	97.46	96.29	97.17	98.09	98.08	97.46	97.27	97.90	97.49	98.30	98.47	97.90
**H2O by diff.**	1.63	1.45	2.41	2.74	2.96	2.90	2.54	3.71	2.83	1.91	1.92	2.54	2.73	2.10	2.51	1.70	1.53	2.10
**Nomenclature**	*Fe-pargasite*	*Actinolite*	*Pargasite*	*Pargasite*	*Edenite*	*Pargasite*	*Edenite*	*Pargasite*	*Actinolite*	*Edenite*	*Actinolite*	*Mg-Hbl*	*Edenite*	*Edenite*	*Mg-Hbl*	*Edenite*	*Actinolite*	*Mg-Hbl*
**T-sites**																		
**Si**	6.32	7.66	6.38	6.40	6.76	6.58	6.59	6.42	7.77	6.61	7.88	7.46	6.85	6.65	7.54	6.51	7.55	6.95
**Aliv**	1.68	0.34	1.62	1.60	1.24	1.42	1.41	1.58	0.23	1.39	0.12	0.54	1.15	1.35	0.46	1.49	0.45	1.05
**Al(total)**	2.41	0.46	2.49	2.46	1.79	2.01	2.09	2.42	0.43	2.09	0.29	0.87	1.69	2.02	0.76	2.18	0.79	1.71
**M1,2,3 sites**																		
**Alvi**	0.73	0.13	0.87	0.86	0.55	0.59	0.68	0.84	0.20	0.70	0.18	0.33	0.54	0.66	0.31	0.69	0.34	0.66
**Ti**	0.04	0.01	0.04	0.04	0.04	0.05	0.04	0.05	0.01	0.04	0.01	0.02	0.04	0.04	0.01	0.05	0.01	0.03
**Fe3+**	0.69	0.21	0.54	0.55	0.60	0.59	0.53	0.45	0.12	0.50	0.04	0.28	0.48	0.49	0.20	0.56	0.13	0.35
**Mg**	2.08	3.21	2.05	2.00	2.47	2.24	2.39	2.17	3.21	2.41	3.42	3.04	2.34	2.39	3.35	2.16	3.11	2.57
**Mn**	0.04	0.03	0.05	0.05	0.08	0.08	0.02	0.02	0.03	0.03	0.04	0.04	0.04	0.02	0.02	0.02	0.02	0.02
**Fe2+**	1.42	1.41	1.45	1.50	1.26	1.45	1.34	1.48	1.43	1.33	1.31	1.29	1.56	1.39	1.11	1.52	1.38	1.36
**Ca**	0.00	0.00	0.00	0.00	0.00	0.00	0.00	0.00	0.00	0.00	0.00	0.00	0.00	0.00	0.00	0.00	0.00	0.00
**Sum M123**	5.00	5.00	5.00	5.00	5.00	5.00	5.00	5.00	5.00	5.00	5.00	5.00	5.00	5.00	5.00	5.00	5.00	5.00
**M4 site**																		
**Fe**	0.10	0.11	0.06	0.06	0.07	0.07	0.05	0.03	0.02	0.04	0.02	0.07	0.04	0.04	0.05	0.06	0.03	0.00
**Ca**	1.66	1.77	1.68	1.69	1.65	1.68	1.71	1.74	1.82	1.70	1.84	1.70	1.69	1.71	1.79	1.70	1.84	1.77
**Na**	0.24	0.12	0.26	0.25	0.28	0.25	0.24	0.23	0.16	0.25	0.14	0.23	0.28	0.25	0.16	0.23	0.13	0.23
**Sum M4**	2.00	2.00	2.00	2.00	2.00	2.00	2.00	2.00	2.00	2.00	2.00	2.00	2.00	2.00	2.00	2.00	2.00	2.00
**A site**																		
**Ca**	0.00	0.00	0.00	0.00	0.00	0.00	0.00	0.00	0.00	0.00	0.00	0.00	0.00	0.00	0.00	0.00	0.00	0.00
**Na**	0.34	0.07	0.33	0.31	0.25	0.31	0.31	0.36	0.04	0.32	0.01	0.09	0.26	0.31	0.07	0.33	0.04	0.15
**K**	0.07	0.02	0.05	0.05	0.04	0.08	0.05	0.07	0.02	0.04	0.01	0.03	0.07	0.05	0.03	0.06	0.03	0.05
**Sum A**	0.41	0.09	0.39	0.36	0.29	0.39	0.36	0.43	0.06	0.36	0.03	0.12	0.33	0.37	0.09	0.39	0.07	0.20
**Sum cations**	15.41	15.09	15.39	15.36	15.29	15.39	15.36	15.43	15.06	15.36	15.03	15.12	15.33	15.37	15.09	15.39	15.07	15.20
**Fe#**	0.52	0.35	0.50	0.51	0.44	0.48	0.44	0.47	0.33	0.44	0.29	0.35	0.47	0.45	0.29	0.50	0.33	0.40
**Mg/Fe2+**	1.37	2.11	1.36	1.29	1.86	1.47	1.72	1.44	2.21	1.75	2.57	2.24	1.46	1.67	2.88	1.36	2.21	1.89
**Mg/Fe**	0.94	1.86	1.00	0.95	1.28	1.06	1.25	1.11	2.05	1.29	2.49	1.86	1.12	1.24	2.46	1.00	2.02	1.50

Table S3. Averages of amphibole chemistry from analyzed samples. Textural context (e.g. cores, mantles, rims) and the number of measurements per sample is reported. Nomenclature of amphiboles follows Leake et al. (1997).

in cores or rims) were used to calculate peak P-T (e.g., if a sample had actinolite cores and hornblende rims, only the hornblende rims were used, see Table S3). We used a plagioclase composition of An3, representing the maximum anorthite component that is typical in low-grade plagioclase before increasing to An18 across the Peristerite gap (Maruyama et al., 1982). This is supported by EDS spectra that show some samples with detectable, albeit extremely small, Ca peaks, but most samples are albite-dominated and Ca is undetectable (which could be a result of retrogression). Results from two approaches that yield values consistent with independent petrologic constraints (e.g., presence of titanite and absence of rutile, presence of albite and absence of jadeite+quartz) are shown in Table S4. The first approach constrains pressures from Schmidt (1992) Al-in-hornblende barometer and calculates temperatures using the Holland and Blundy (1994) thermometer. The second is an iterative approach of Anderson and Smith (1995), which accounts for the effects of oxygen fugacity on the Al-in-hornblende barometer. Using a plagioclase composition of An1 reduces temperatures by ~50˚C for the first approach (Schmidt P first). For the iterative approach, An1 yields pressures that are ~0.4-1 kbar lower and temperatures ~60˚C colder. These estimates can be refined further by quantifying plagioclase chemistry.

^ Uncertainty in Schmidt (1992) Al-in-Hbl barometer is reported as ± 0.6 kbar
* Uncertainty in temperature estimates is reported as ±75˚C for BH90, and ±40˚C for HB94

**Texture**	*cores*	*foliation*	*oblique*	*rims*	*mantles*	*cores*	*rims*	*cores*	*unzoned*	*rims*
**Sample ID**	*AK219*	*AK222*	*AK222*	*AK243*	*AK253*	*AK264*	*AK268*	*AK275*	*AK278*	*AK295*
**# Analyses**	*30*	*37*	*14*	*16*	*5*	*51*	*19*	*14*	*58*	*3*
**Nomenclature**	*Fe-Parg*	*Pargasite*	*Pargasite*	*Pargasite*	*Pargasite*	*Edenite*	*Edenite*	*Edenite*	*Edenite*	*Mg-Hbl*
**Results based on Schmidt pressure (used for purposes of calculation)**										
**Pschmidt (kb)**	**8.5**	**8.8**	**8.7**	**6.6**	**8.5**	**6.9**	**5.0**	**6.6**	**7.4**	**5.1**
T (C) HB1* '94	646	624	624	628	615	610	605	610	626	559
**T (C) HB2^ '94**	**534**	**509**	**504**	**516**	**505**	**494**	**486**	**494**	**511**	**434**
T (C) BH '90	680	665	664	662	663	651	635	650	664	615
**Results based on iteration using Anderson and Smith pressure at various thermometers**										
T (C) HB1*	648	629	628	628	620	612	605	612	628	560
P(Kb) HB1*	8.8	9.4	9.3	7.0	9.2	7.5	5.5	7.1	7.9	5.5
**T (C) HB2 ^**	**542**	**518**	**512**	**519**	**513**	**497**	**485**	**496**	**516**	**423**
**P(Kb) HB2 ^**	**9.4**	**9.7**	**9.5**	**7.0**	**9.3**	**7.3**	**4.8**	**6.8**	**7.9**	**3.9**
T (C) BH	682	662	660	659	660	647	630	646	661	609
P(Kb) BH	8.4	9.0	8.9	6.7	8.7	7.2	5.4	6.9	7.5	5.6

* HB 1 refers to Holland and Blundy Hbld-Plag thermometry calibration reaction edenite + 4 quartz = tremolite + albite
^ HB 2 refers to Holland and Blundy Hbld-Plag thermometry calibration reaction edenite + albite = richterite + anorthite
BH refers to Blundy and Holland Hbld-Plag thermometry calibration reaction edenite + 4 quartz = tremolite + albite

Table S4. Results from multi-equilibrium P-T calculations based on co-stable amphibole and plagioclase compositions during peak conditions (i.e., inverse thermobarometry). Number of analyses indicates the number of microprobe measurements that were averaged to produce the chemical compositions in Table S3. Grey rows are preferred results from each method; HB2 calibration is preferred. See Anderson (1996) for details.

Text S4. U-Pb titanite analyses and age calculations

All laser spots were carefully screened after analysis to remove erroneous data, e.g. accidental analysis of allanite grains, laser pits that were off-target (see Fig. S5 for supplemental images and EDS spectra from post-analysis characterization).

Our decision to discretize our titanite U-Pb data into 3-sec ablation increments, rather than using the bulk integration, lowers the uncertainty and improves the regression precision. However, we emphasize here that this is not artificial, but attributable to several analytical aspects:

1. The approach allows us to more effectively exclude outliers from the data in a less biased fashion than manual adjustments (e.g., related to inclusions encountered during ablation).
2. The approach does not artificially degrade the analytical uncertainty to lower the MSWD. While this might be true for 1 sec depth-profile increments with much larger counting analytical uncertainties than the bulk data, this is not true for 3-sec increments. The 3-sec increments do not have drastically larger uncertainties and thus ensure that the overall increment uncertainty remains robust. In fact, in a number of cases, the 3-sec increment uncertainty is improved due to the exclusion of aberrant data (see first point regarding inclusions and other data outliers). Hence, the 3-sec data increment precision is NOT artificially degraded. Increases in MSWD and dispersion in both bulk and incremental data appears to mainly stem from very low U titanite, suggesting that variations in common Pb composition – a variation likely attributable to small non-radiogenic inclusions – are responsible.
3. As articulated in the manuscript, the fundamental reason for breaking the bulk analysis into 3-sec increment is to leverage small and significant variations in U and Pb common lead content during depth profiling. While 1-sec

increments would also accomplish this, individual increment uncertainties would be very large and indeed artificially degrade the uncertainty. As previously pointed out, these systematic variations increase the spread along the radiogenic-common Pb mixing line and hence improve the regression precision – a common practice in all isochron methodologies. This increase in regression precision is not artificial. In fact, the variations in U and common Pb are real and can be seen in the raw and 3-sec incremental data and this is what we are leveraging. In contrast, bulk data tend to homogenize and obscure these variations and hence reduce the spread along the Tera-Wasserburg mixing line and increase the regression uncertainty. With that said, we do not know where these variations in the U and common Pb in the titanite crystals are related to intra-crystalline heterogeneities, growth zones, or mineralogical/crystallographic intricacies. Given their magnitudes of the variations, however, these are likely not inclusions.

Secondary standards (BLR) were plotted in Tera-Wasserburg space and anchored to 207/206 Pb value of 0.91 (Sun et al., 2012). The lower intercept for sample 203 is consistent with the standard age of BLR (~1030 Ma). For sample 209, anchoring the BLR secondary standards yields a lower intercept that is significantly older (~1080 Ma). BLR standard ratios do not cluster between runs, suggesting our reported errors for sample 209 may be underestimated, however we emphasize that data are internally consistent for a given run.

Table S4 shows the different age calculations from U-Pb data from bulk (whole grain) analyses and 3-second integrated analyses. Data were filtered and regressed according to microstructure (e.g., see 'fabric' column) and [U]. The ages regressed from bulk data derived from grains that define the S1 and S2 fabrics overlap within error of the 'preferred' age presented in the main text (90.8 $\pm$ 2.5 Ma). We consider the 3-second integrated data a more robust result due to greater spread in [U] and a slightly better MSWD. Note that the ages calculated from S2 grains only are slightly older, but still overlap within error with the final reported age. For this sample, the strictness of the [U] filter does not severely impact calculated ages; even the strictest $\geq$10 ppm filter overlaps with the ~91 Ma age within error and has a good MSWD.

For sample 209 regressions from bulk grain data are geologically insignificant (as far as sole metamorphism goes) and clearly do not overlap with our reported age of ~90 $\pm$ 6 Ma. This could point to mixing between different generations of titanite, but without higher-resolution isotopic/geochemical analysis and imaging, we prefer not to put weight on this interpretation. Ages regressed from integrated 3-second data have tighter uncertainties and better MSWDs, generally speaking. Similar to sample 203, different [U] filters yield similar ages that overlap within error, spanning ~ 95-90 Ma. We selected the least restrictive [U] for our preferred age since this sample has less U and the 2 ppm filter yields a similar number of data points as the regression for sample 203.

Figure S5. (Next page) Supplemental BSE images and examples of EDS spectra from dated titanite-bearing samples. (A-D) Sample AK203; (A) S1 is refolded to form an S2 cleavage and is locally affected by (B) weak crenulation that is non-penetrative in AK203. Most titanites are syn S1-S2. (C) S2 cleavage defined by well-aligned allanite grains (see Spectra 14,17); these were removed from the U-Pb regression. (D) Lazed titanites and one allanite grain (removed from regression). Note that unlazed grain spectra 14 is a clean, inclusion-free, unzoned titanite. (E-F) Sample AK209. S3 cleavage oriented from top right to bottom left of the image; most titanites are syn-S3. All dated grains have been carefully screened, their identities (titanite vs. allanite) confirmed with EDS, and allanite data has been removed from the U-Pb regressions.

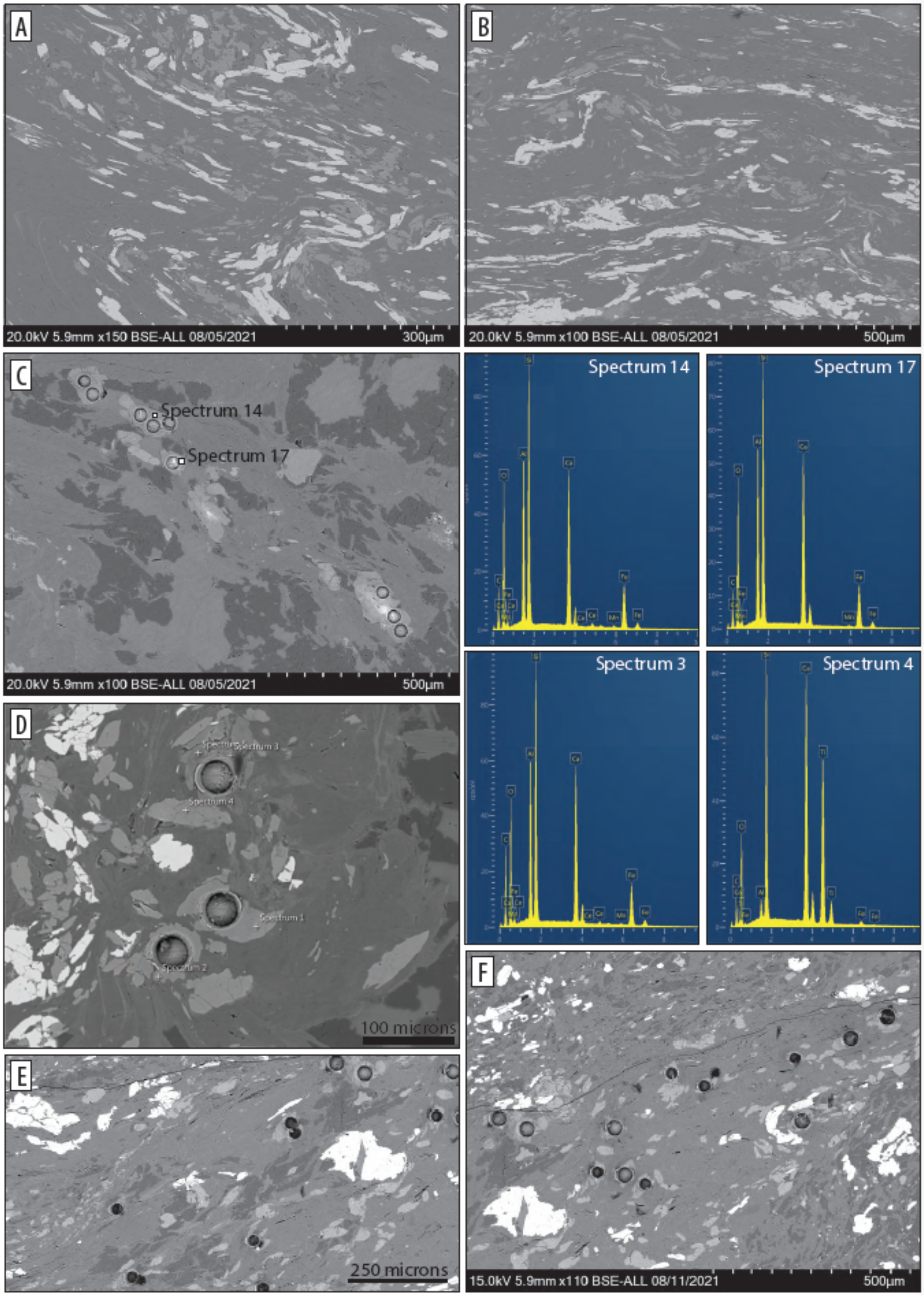
A
20.0kV 5.9mm x150 BSE-ALL 08/05/2021
300µm
B
20.0kV 5.9mm x100 BSE-ALL 08/05/2021
500µm
C
Spectrum 14
Spectrum 17
20.0kV 5.9mm x100 BSE-ALL 08/05/2021
500µm
Spectrum 14
Spectrum 17
D
100 microns
Spectrum 3
Spectrum 4
E
250 microns
F
15.0kV 5.9mm x110 BSE-ALL 08/11/2021
500µm

Calculated U-Pb ages from titanite grains in LT sole samples. Results from different data processing and filtering techniques are shown. Uncertainties on U-Pb age calculations are calculated from IsoplotR, where x is the analytical uncertainty (sigma) of the U-Pb age and y is the 100(1-a)% confidence interval. For bulk ages, n = number of analyzed grains. For integrated ages, n = number of 3-second data slices.

Sample	Data type	Fabric	n	[U] filter	207Pb/206Pb	± x	Age	± x	± y	MSWD
AK-82Z-03-202.9	Bulk	all	83	none	0.836	0.003	95	1.6	3.3	1.6
	Bulk	all	53	≥ 5 ppm	0.832	0.003	94.1	1.7	3.2	1.7
	Bulk	S1 & S2	53	none	0.833	0.004	91.8	3.0	5.9	1.6
	Bulk	S1 & S2	36	≥ 5 ppm	0.825	0.004	88.1	3.2	6.2	1.4
AK-82Z-03-202.9	Integrated	all	438	≥ 5 ppm	0.838	0.003	95.9	1.4	2.7	1.3
	Integrated	S1 & S2	495	none	0.836	0.003	93.5	2.4	4.7	1.4
	**Integrated**	**S1 & S2**	**284**	**≥ 5 ppm**	**0.830**	**0.004**	**90.8**	**2.5**	**4.9**	**1.3**
	Integrated	S1 & S2	228	≥ 6 ppm	0.824	0.004	89.6	2.6	5.7	1.3
	Integrated	S1 & S2	115	≥ 10 ppm	0.829	0.006	91.7	3.0	6.0	1.3
	Integrated	S2 only	208	≥ 5 ppm	0.834	0.004	95.4	3.4	6.6	1.4
	Integrated	S2 only	171	≥ 6 ppm	0.830	0.004	94.5	3.5	6.8	1.3
AK-86Z-04-209.35	Bulk	all	74	none	0.857	0.005	121.3	11.3	22.1	1.9
	Bulk	all	12	≥ 5 ppm	0.834	0.013	108.5	18.4	36.0	1.2
	Bulk	S3 only	45	none	0.857	0.007	123.4	13.0	25.5	1.9
AK-86Z-04-209.35	Integrated	all	418	≥ 2 ppm	0.844	0.004	90.7	5.3	12.4	1.4
	Integrated	all	117	≥ 5 ppm	0.842	0.007	93.2	6.2	12.2	1.1
	**Integrated**	**S3 only**	**254**	**≥ 2 ppm**	**0.842**	**0.005**	**90.2**	**5.7**	**13.7**	**1.5**
	Integrated	S3 only	118	≥ 4 ppm	0.849	0.007	95.4	6.4	12.5	1.2
	Integrated	S3 only	78	≥ 5 ppm	0.849	0.008	95.1	6.6	13.0	1.1

Table S5. Results from using bulk vs. 3-second integrated titanite analyses for U-Pb ages.

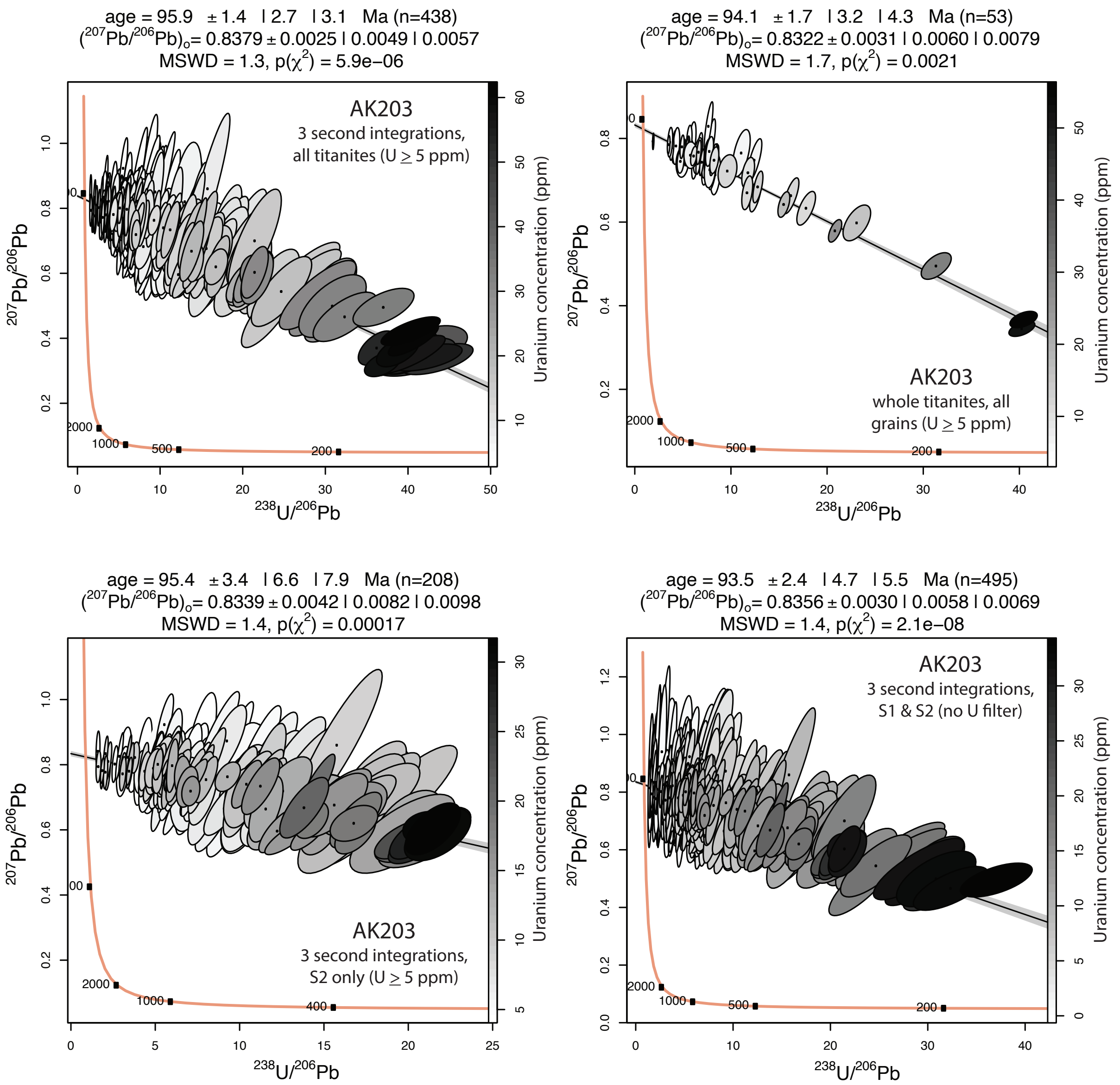

Figure S6. Supplemental Tera-Wasserburg plots showing results of different data filtering for sample 203. Whole titanites and 3-second integrated data are shown. Selected from Table S4.

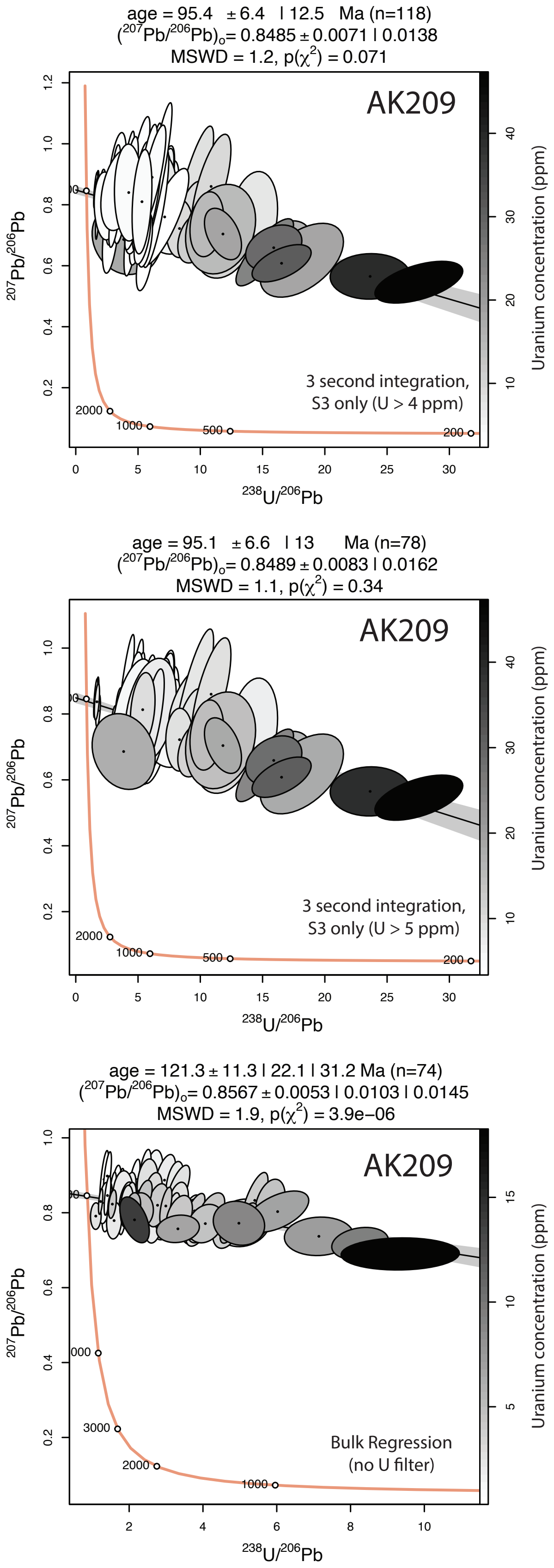

Figure S7. Supplemental Tera-Wasserburg plots showing results of different data filtering for sample 203. Whole titanites and 3-second integrated data are shown. Selected from Table S4.

Data Set S1. All XRD analyses from visually distinct compositional layers, and foliation-parallel and cross-cutting veins.

Data Set S2. All titanite U-Pb analyses (bulk and 3-second integrated data) from samples 203 and 209 and standards.
